# The immune factors have complex causal regulation effects on bone mineral density

**DOI:** 10.3389/fimmu.2022.959417

**Published:** 2022-10-20

**Authors:** Rong-Rong Cao, Xing-Hao Yu, Meng-Fei Xiong, Xue-Ting Li, Fei-Yan Deng, Shu-Feng Lei

**Affiliations:** ^1^ Center for Genetic Epidemiology and Genomics, School of Public Health, Medical College of Soochow University, Suzhou, China; ^2^ Jiangsu Key Laboratory of Preventive and Translational Medicine for Geriatric Diseases, Soochow University, Suzhou, China

**Keywords:** immune cells, osteoimmunology, Mendelian randomization, bone mineral density, osteoporosis

## Abstract

Recent evidence has gradually recognized that the immune and skeletal systems are two closely correlated systems, but the specific immune factors on bone mineral density (BMD) are largely unknown. Based on the summary-level data of genome-wide association studies (GWASs), we performed a series of analyses including two-sample Mendelian randomization (MR) analysis to test potential causal links between 731 immune traits [including median fluorescence intensities (MFIs), absolute cell (AC) counts, relative cell (RC) counts, and morphological parameters (MP)] and BMD. After false discovery rate (FDR) correction, 9 MFI-BMD, 16 AC-BMD, 22 RC-BMD, and 5 MP-BMD pairs reached the level of significance (FDR-adjusted *p*< 0.05). For MFI traits, the T- and B-cell panels had the largest number of significant immune trait pairs than other panels. CD40, as a molecule expressed by four subsets of monocytes, was highlighted due to its consistently positive correlation with BMD at four sites. For both AC and RC traits, immune traits from the T-cell panel were also highlighted, with CD39-positive T-cell subsets being the most frequently observed feature. For MP traits, the most significant association immune trait with BMD was SSC-A on CD14^+^ monocyte. Sensitivity analyses suggested that the identified immune factors were robust to pleiotropy. Multivariable MR analysis confirmed the independent causal effect of several immune traits on BMD. Mediation analyses showed that CD40 on monocytes could mediate multiple immune traits, especially the suggestive associations of CD27 on several memory B cells with BMD mediated by CD40 on CD14^+^ CD16^−^ monocyte. Our study represents the first comprehensive evaluation of the causal effects of immune traits on the risk of osteoporosis. The findings highlighted the complex and important role of immune-derived factors in the pathogenesis of osteoporosis.

## Introduction

Osteoporosis is a common multifactorial bone disease characterized by low bone mass and microarchitectural deterioration of bone tissue resulting from increased bone resorption and impaired bone formation (1). The reduced bone mass leads to increased susceptibility to fracture (2). Worldwide, it is estimated that by 2040, the number of osteoporotic patients over 50 years old will double from 158 million in 2010 (3). The burden of osteoporosis is projected to reach $25.3 billion by 2025 in the USA alone (4). The pathogenesis of osteoporosis is extremely complex and largely unknown (5, 6).

Recent evidence has gradually recognized that the immune and skeletal systems are two closely correlated systems, and immune dysregulation plays an important role in osteoporosis pathogenesis, but the specific immune factors are largely unknown. The maintenance of bone homeostasis depends on the dynamic balance of cellular activities during bone remodeling. It is widely accepted that the variety of molecular and cellular factors involved in maintaining and increasing bone mass is far more numerous than originally anticipated. “Osteoimmunology” is a new emerging but rapidly developing interdisciplinary term. It summarizes the progress of complex interplay between the immune and skeletal systems. Recent studies in this field have made much progress in the understanding of such complex interplay, especially the effects of the immune system on osteoclasts (7). Indeed, osteoclasts derived from the common myeloid progenitors (CMPs) and adaptive immune cells originated from the common lymphoid progenitors (CLPs) share a common origin, i.e., multipotent progenitors (MPPs), which are differentiated from multipotential hematopoietic stem cells (HSCs) that reside in an immunological organ—the bone (8). In particular, due to the progress in the pathogenesis of osteoporosis, the field of osteoimmunology has developed a new branch named “immunoporosis” (9). Primary osteoporosis caused by aging and estrogen deficiency or secondary osteoporosis caused by autoimmune diseases or infections are all induced or aggravated by aberrant immune activation. All these conditions are paralleled by a proinflammatory response state and the presence of nuclear factor (NF)-κB ligand (RANKL) producing activated T cells. Undoubtedly, RANKL expressed by osteoblasts, osteocytes, activated T cells, and B cells is one of the most critical cytokines explicitly linking the two systems, which is necessary and sufficient to regulate the differentiation and proliferation of osteoclast precursors into mature multinucleated osteoclasts by binding to its receptor RANK on osteoclasts and their precursors (10–13). Furthermore, evidence from *in vitro* studies has shown that osteoclast differentiation under pathological conditions is also regulated by a host of immune factors including costimulatory receptors, cytokines, and immune cells such as T and B lymphocytes (14, 15). Recent studies in human populations have highlighted the role of immune factors in the development of osteoporosis. Increased levels of pro-osteoclastogenic factors such as tumor necrosis factor-alpha (TNF-α) and RANKL and more granulocyte macrophage colony-stimulating factor (GM-CSF) secreted by B lymphocytes or decreased levels of anti-osteoclastogenic factors [e.g., interferon-gamma (IFN-γ)] were associated with osteoporosis risk (16–21). The above-described findings have only confirmed that close connections exist between osteoporosis and the immune system, but the specific immune factors underlying such connections are still largely unknown.

Recently, Mendelian randomization (MR) is a powerful and effective analysis that uses genetic variants as instrumental variables (IVs) or proxies to evaluate the causal relationship between variable and outcome (22). Genetic variation from parent to offspring is randomly assigned at the time of gametogenesis, protecting the genotype–phenotype link from the bias introduced by confounding factors seen in observational studies and reverse causation (23).

Motivated by the above findings, this study performed this first comprehensive two-sample Mendelian randomization analysis to assess the causal links between immune traits and osteoporosis. Although some causal links supported by previous functional studies were found in our study, many more novel associations that have no previously supported evidence were detected. These findings greatly increase the understanding of the connections between the two systems and provide an appropriate framework for how different immune factors influence the development of osteoporosis. The results can also provide helpful clues for future functional studies.

## Materials and methods

### GWAS data sources

In order to obtain a more comprehensive and reliable conclusion of the causal relationship between immune indicators and BMD, we selected the largest GWAS published to date for immunophenotyping of peripheral blood, of which 118 were absolute cell (AC) counts, 389 were median fluorescence intensities (MFIs) reflecting the levels of surface antigens, 32 were morphological parameters [MP, forward scatter (FSC) and side scatter (SSC), which are proportional to the cell volume, and intracellular complexity and the surface texture of cells, respectively], and 192 were relative cell (RC) counts. The GWAS summary statistics for 731 immune traits could be publicly available in the GWAS Catalog (accession numbers from GCST0001391 to GCST0002121) (24). This GWAS analysis was performed based on 3,757 Sardinian samples (57% women) to test around 22 million single nucleotide polymorphisms (SNPs) genotyped with high-density arrays after adjusting for sex, age, and age^2^. The SNPs were imputed with a Sardinian sequence-based reference panel (25). The outcomes include four DXA-BMD phenotypes [i.e., total body (TB-) BMD, lumbar spine (LS-) BMD, forearm (FA-) BMD, femoral neck (FN-) BMD], and GWAS summary statistics of European participants could be downloaded from Genetic Factors for Osteoporosis (GEFOS) Consortium (http://www.gefos.org/) (26, 27). Summary statistics for life course TB-BMD were generated after testing ~23,700,000 SNPs based on 30 GWAS cohorts with 66,628 European individuals, which were further divided into five age stages (each spanning 15 years). Genotypes were imputed by using the 1000 Genomes Project. Summary statistics for LS-BMD, FN-BMD, and FA-BMD were obtained after adjusting for sex, age, age^2^, and weight. The detailed information (e.g., effect size, standard error, and sample size) for each SNP was reserved for further analysis, and we summarized the information of all data sets in **Table 1** and **Supplementary Table S1**.

**Table 1:** Summary information for the genetic data used in the present study.

Phenotypes		Year	Sample size (case/control)	m	PMID
Immune cell traits		2020	3,757	20,143,392	32929287
Life course TB-BMD	0–15 years	2018	11,807	10,026,671	29304378
	15–30 years		4,180	9,260,665	
	30–45 years		10,062	10,343,119	
	45–60 years		18,805	11,563,715	
	>60 years		22,504	13,486,402	
	All individuals		66,628	15,206,491	
DXA-BMD	Femoral neck	2015	32,735	9,890,024	26367794
	Lumbar spine		28,498	9,890,447	
	Forearm		8,143	9,391,221	

TB-BMD, total body bone mineral density; DXA, dual-energy X-ray absorptiometry; m, the number of genetic variants.

### Selection of instrumental variables

In accordance with data used in the recent research (24), multiple instrumental variables (IVs) were selected for each immune trait with the same criteria in our MR analysis. The significant IVs were determined with a loose threshold of 1.00E−5. Then, PLINK software (version v1.90) was used to prune these IVs, where linkage disequilibrium (LD) r^2^ was calculated in 1000 Genomes Projects (28). The LD threshold r^2^ threshold was set to 0.1, and the physical distance was set to within 500 kb. Additionally, for BMD traits, we set a stricter threshold with a significant threshold of 5.00E−8 and r^2^ of 0.01. To avoid weak instrumental bias in MR analysis, the proportion of phenotypic variation explained (PVE) was calculated, and then, the F statistic of instruments for each immune trait was used to evaluate the strength of IVs. Typically, IVs with low F statistics (<10) were removed from our analysis. Following a uniform procedure, we obtained a total of 7–1,786 independent IVs with a loose significance for further analysis. Then, those SNPs were extracted from each BMD trait, respectively. On average, these generated IVs could explain 0.240% (range, 0.004%–3.652%) of the variance in their respective immune traits. The strength of IVs was estimated in terms of F statistic and ranged between and for immune traits, and the detailed information for each trait is illustrated in **Supplementary Table S1**.

### Estimation of causal effect and sensitivity analysis

To evaluate whether immune traits have causal effects on the risk of osteoporosis, several robust analytical MR methods based on different assumptions were utilized to estimate the causal associations between variables and outcome phenotypes, including fix-effect inverse variance weighting (IVW) (29), random-effect IVW (29) as the main analyses, with sensitivity analyses using weighted median-based, MR-Egger (30), and MR pleiotropy residual sum and outlier (MR-PRESSO) (31). Initially, the IVW method was conducted as the primary analysis, based on which we separately performed a false discovery rate (FDR) correction (Benjamini and Hochberg) for nominally significant associations between immune traits (with a total of 28 subgroups from 4 types comprising 7 panels) and BMD to control for the proportion of false positives in multiple testing. Additionally, we performed multiple comprehensive sensitivity analyses to rule out the possible violations of the MR assumptions (i.e., heterogeneity and pleiotropy). Heterogeneity was tested by Cochran’s Q statistic (32), a *p*-value of<0.05 would be regarded as significant heterogeneity. If the null hypothesis is rejected, indicating possible heterogeneity within IVs, then random effect IVW is used instead of fixed effect IVW (29). The presence of pleiotropy was detected with intercept item in MR-Egger regression and MR-PRESSO global test. Finally, the Cook’s distance test and MR-PRESSO outlier test were conducted to explore the possibility of bias in the MR results due to pleiotropy (31). After eliminating IVs with pleiotropic effects, we repeated the main MR analysis. To examine whether there exists a causal effect of osteoporosis on immune traits, reverse MR analysis was additionally performed based on the IVs generated based on summary statistics from BMD GWAS.

### Multivariable and mediation MR analysis

A novel multivariable MR analysis was conducted to identify the independent immune cell traits associated with BMD after controlling for other traits due to the possible horizontal pleiotropy (33, 34). SNPs associated with these traits were used as IVs, and their corresponding information, and that of BMD, was eventually incorporated into our MR framework:




$${\hat{\lambda }}^{BMD}\;=\;{\hat{\lambda }}_{trait_{i}}\beta_{trait_{i}}+L+{\hat{\lambda }}_{trait_{j}}\beta_{trait_{j}}\;+\;e,e∼N(0,\sigma^{2})$$

(34) where $\hat{\lambda }$ represents marginal effect size of instruments, *σ* ^2^ represents the variance for residual term *e*, and *β*_*trait*_*i*__ represents the causal effect of two immune cell traits on BMD, respectively. Then, we estimate the effect size of *β*_*trait*_*i*__ and *β*_*trait*_*j*__ with the weighted least squares method. Based on the multivariable MR framework, we calculated the direct and indirect effects of traits on BMD *via* the monocyte (33, 35). Notably, unlike multivariable MR analysis, we only used SNPs related to the trait that serves as an instrumental variable (34). Finally, the confidence intervals of indirect and direct effects were estimated by using bootstrap methods with 1,000 replications.

## Results

### Overview

**Figure 1:**
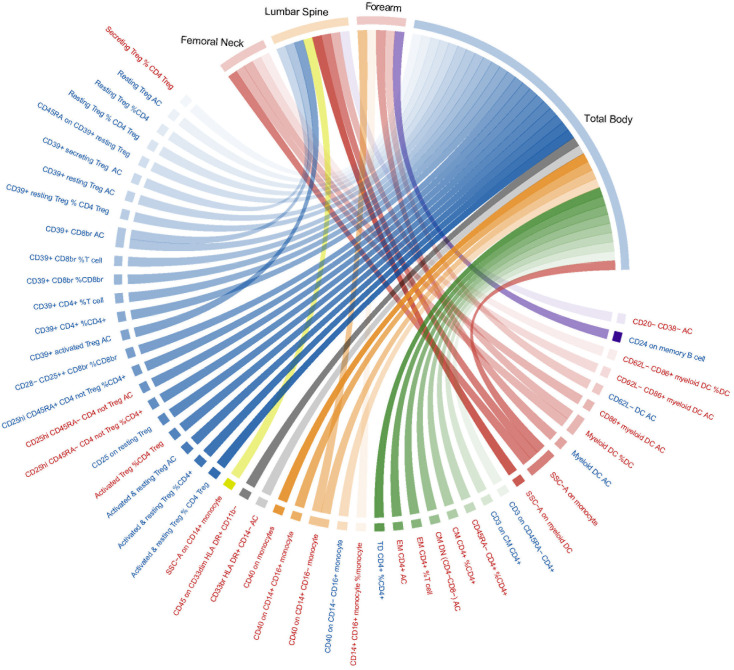
Mendelian randomization associations of immune traits (MFI, AC, RC, and MP) on BMDs that derived from the IVW analysis after FDR correction. Immune traits positively associated with BMD are shown in red, while those negatively associated with BMD are shown in blue. IVW, inverse-variance weighted; BMD, bone mass density; MFIs, median fluorescence intensities; AC, absolute count; RC, relative count; MP, Morphological parameter; FDR, the false discovery rate.

**Supplementary Table S2** lists all the MR analysis results that reached a nominal significance (*p*< 0.05) for a total of 218 pairs between 731 immune traits and outcome (BMD at any site). Given that immune traits were divided into four types of phenotypes (MFI, AC, RC, and MP) including seven panels of immune cells, the detected potential immune signatures associated with BMD can be summarized into 132 MFIs, 53 ACs, 85 RCs, and 13 MP traits (with some overlapping signals shared by BMDs at the four sites) (**Supplementary Table S2**). When immune traits were categorized according to the 7 panels, 37 traits belonged to the maturation stages of T cell, 27 from TBNK, 80 belonged to Treg, 58 classified as B cell, 25 from Myeloid cell, 36 belonged to cDC, and 20 within monocyte were found to be suggestively associated with BMD. Considering the possible false positives, we used the FDR method to correct the *p*-values in different trait types and panels. Among them, only two MFI/BMD trait pairs were found to be not robust in sensitivity analysis and were eliminated (**Supplementary file**). After multiple test corrections and sensitivity analysis, 53 immune trait/BMD pairs, including 9 MFIs (with one trait shared by TB- and FA-BMD), 16 ACs, 22 RCs, and 5 MPs, remained statistically significant (FDR-adjusted *p*< 0.05) (**Figure 1**). Briefly, there were multiple significant associations with BMD based on either the four major types of immune traits or the seven panels of immune cells subdivided by them. Herein, we summarized the complex results into four parts according to immune phenotype types.

### Median fluorescence intensities and BMD

**Supplementary Figure S1** shows that 132 pairs between MFI/BMD reached suggestive association (*p*< 0.05) by using IVW MR analysis. The T-cell panel (e.g., Treg and TBNK) and B-cell panel both had the largest number of significant associations than other panels. CD25 was the most frequently observed molecule expressed in different types of immune cells. In addition, CD40 on CD14^+^ CD16^+^ monocyte trait was the most significant trait compared with other traits in this part (*p* = 1.31E−06).

**Figure 2:**
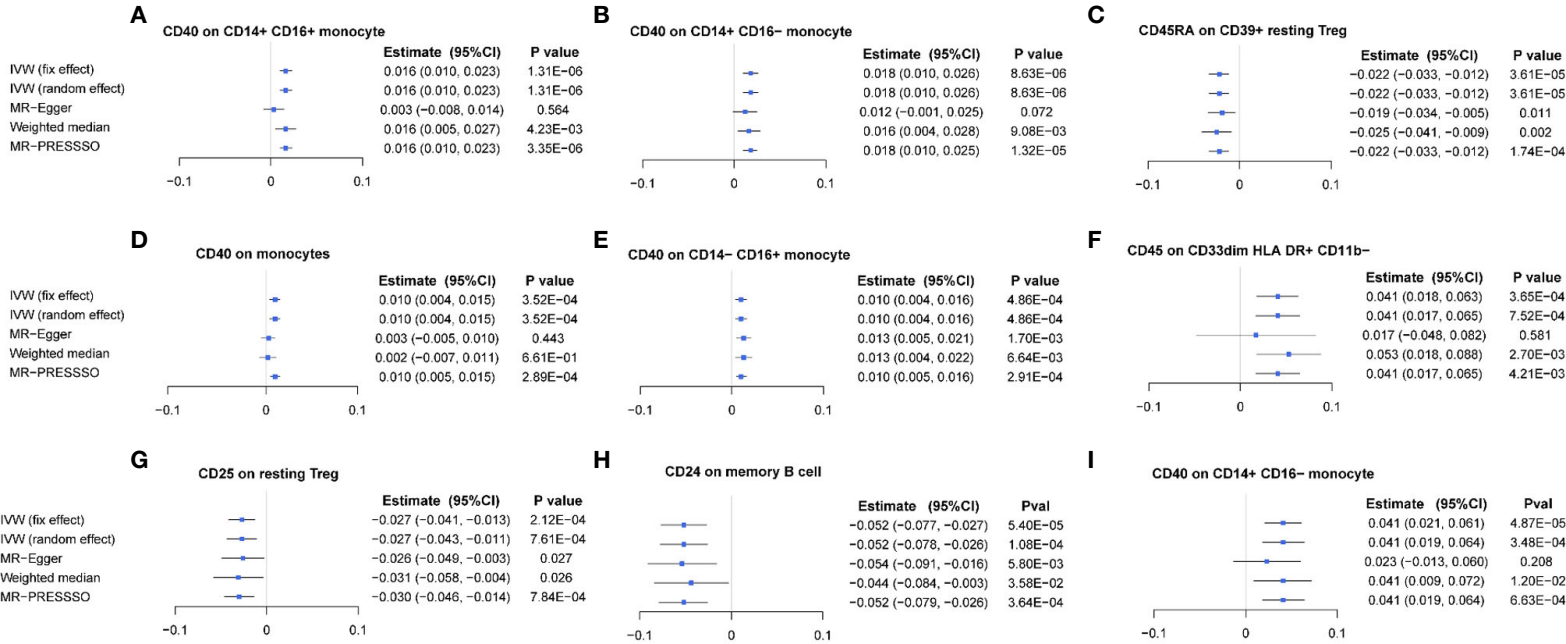
Forest plot of causal effects of different immune traits (MFI) on BMDs. **(A)** CD40 on CD14^+^ CD16^+^ monocyte on TB-BMD. **(B)** CD40 on CD14^+^ CD16^-^ monocyte on TB-BMD, **(C)** CD40 on CD14^−^ CD16^+^ monocyte on TB-BMD, **(D)** CD40 on monocytes on TB-BMD, **(E)** CD45RA on CD39^+^ resting Treg on TB-BMD, **(F)** CD25 on resting Treg on TB-BMD, **(G)** CD45 on CD33dim HLA DR^+^ CD11b^−^ on TB-BMD, **(H)** CD24 on memory B cell on FA-BMD, **(I)** CD40 on CD14^+^ CD16^−^ monocyte on FA-BMD. MFIs, median fluorescence intensities, TB, total body; FA, forearm; BMD, bone mineral density.

**Figure 3:**
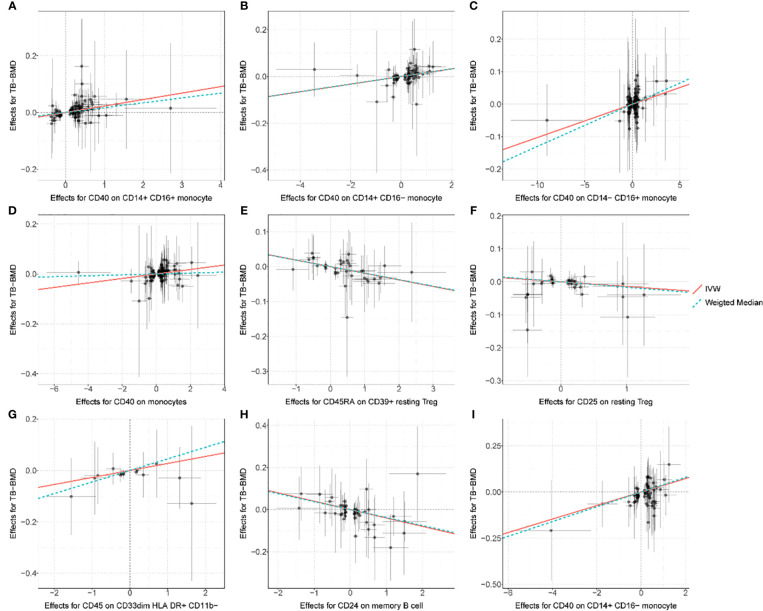
Scatter plot for the relationship between the SNP effect size of causal immune traits (x-axis) and the corresponding effect size estimates of BMDs (y-axis). **(A)** CD40 on CD14^+^ CD16^+^ monocyte on TB-BMD. **(B)** CD40 on CD14^+^ CD16^−^ monocyte on TB-BMD, **(C)** CD40 on CD14^−^ CD16^+^ monocyte on TB-BMD, **(D)** CD40 on monocytes on TB-BMD, **(E)** CD45RA on CD39^+^ resting Treg on TB-BMD, **(F)** CD25 on resting Treg on TB-BMD, **(G)** CD45 on CD33dim HLA DR^+^ CD11b^−^ on TB-BMD, **(H)** CD24 on memory B cell on FA-BMD, **(I)** CD40 on CD14^+^ CD16^−^ monocyte on FA-BMD. TB, total body; FA, forearm; BMD, bone mineral density.

After FDR correction, the IVW MR analyses identified nine significant MFI/BMD trait pairs (FDR-adjusted *p*< 0.05) (**Supplementary Table S3**, forest plot in **Figure 2**, scatter plot in **Figure 3**, and funnel plot in **Supplementary Figure S2**). Among them, up to four traits (with one trait, CD40 on CD14^+^ CD16^−^ monocyte, shared by TB- and FA-BMD) were from the monocyte panel, suggesting the potential importance of monocyte on the bone. In addition to the MR-Egger regression method, the major IVW and other MR analyses provided supporting evidence for the causal effects of four monocyte-related MFI traits on BMD (**Figure 2**). Furthermore, CD40 was highlighted as a molecule expressed by four subsets of monocytes with consistently positive correlations with BMD **(** **Supplementary Table S3** **)**. In addition, as shown in the scatter plot (**Figure 3**), the trait (i.e., CD45 on CD33^dim^ HLA DR^+^ CD11b^−^) was positively associated with BMD, while the traits (e.g., CD24 on memory B cell, CD25 on resting Treg, CD45RA on CD39^+^ resting Treg) were negatively associated with BMD (**Figures 3E–G**).

### The absolute cell counts and BMD

The suggestive significances of 53 pairs between AC traits and BMD are shown in **Supplementary Figure S3** and **Supplementary Table S4** (*p*< 0.05). Similarly to the findings in MFI, the traits from the T-cell panel had the highest number of significant associations compared to those from the other panels, in which the most frequent association signal was observed for CD39-positive T-cell subgroups (e.g., CD39^+^ activated/secreting Treg AC, CD39^+^ CD8^br^ AC and CD39^+^ CD4^+^ AC). In addition, CD39^+^ resting Treg AC had the highest level of significance (*p* = 5.96E−06). After FDR correction, 16 AC traits had significant causal effects on BMD estimated from different MR methods (FDR-adjusted *p*< 0.05) (**Supplementary Table S4**). In this section, CD39-positive T-cell subsets presented the most frequently observed significant associations with BMD. Moreover, for these most significant pairs, no heterogeneity was detected by sensitivity analysis (**Supplementary Figure S4**), and consistent association signals were observed by using different MR methods (**Supplementary Figure S5**). As shown in **Supplementary Figure S6**, positive associations were revealed between such as effector memory (EM) CD4^+^ (β = 0.021, *p* = 1.93E−05), CD33^br^ HLA DR^+^ CD14^−^ (β = 0.008, *p* = 2.00E−03), CD25^hi^ CD45RA^−^ CD4 not Treg (β = 0.013, *p* = 1.98E−03), central memory (CM) DN (CD4^−^CD8^−^) (β = 0.027, *p* = 8.62E−04), and BMD. On the other hand, the negative associations between the other traits (e.g., CD39^+^ resting Treg, Resting Treg, activated and resting Treg, CD39^+^ CD8^br^) and BMD were also detected.

### The relative cell counts and BMD

A total of 85 RC traits/BMD pairs were detected at the significant level of *p*< 0.05 (**Supplementary Figure S7**). Among them, the traits from the T-cell panel were still highlighted, as the proportion of detected significant pairs was as high as the proportion of 77.65% (66 T-cell pairs *vs*. 85 total pairs). In particular, a considerable number of CD39-positive traits were co-associated with at least two of the BMDs in Treg panel. The strongest significant association (*p* = 3.17E−05) of CD39^+^ resting Treg %CD4 Treg (the percentage of CD39^+^ resting Treg with respect to one of its parent cell lineages, CD4 Treg) with BMD was observed, which further suggested that such cells play an important role in OP progression.

After FDR correction, 22 RC traits were significantly associated with BMD (**Supplementary Table S4**). Among them, the traits from the T-cell panel were still highlighted, especially from the CD39-positive T cells. No significant heterogeneity existed for these most significant pairs (**Supplementary Figure S8** **)**. The forest plot (**Supplementary Figure S9** **)** demonstrated that the association results were almost consistent by using several MR analyses. As presented in **Supplementary Figure S10**, some CD39-positive T cells were negatively associated with TB-BMD (e.g., CD39^+^ resting Treg %CD4 Treg, CD39^+^ CD4^+^ %CD4^+^, CD39^+^ CD4^+^ %T cell, CD39^+^ CD8^br^ %T cell, and CD39^+^ CD8^br^ %CD8^br^). However, the reverse MR analysis suggested some reverse causal effect of TB-BMD on CD39^+^ CD4^+^ %T cell (**Supplementary Table S8**).

Interestingly, there were several subpopulations (resting Treg, CD39^+^ resting Treg, activated and resting Treg, secreting Treg, and activated Treg) that represent the different activation statuses of CD4^+^ Treg cells. The RC parameters for these subgroups were consistently and significantly associated with BMD (**Supplementary Table S4**). Among them, activated and secreting Treg cells appeared to have a protective effect against osteoporosis compared to resting Treg cells.

As to the differentiation status of T cells, the IVW estimates provided strong evidence for positive effects of CM CD4^+^ %CD4^+^ and EM CD4^+^ %T cell and a negative effect of terminally differentiated (TD) CD4^+^ %CD4^+^ on BMD. Remarkably, these links between EM CD4^+^ %T cell and BMD were mostly concordant with findings of its progenitor cell lineages in AC traits. For more information on MR results for traits in other panels associated with BMD, see **Supplementary Table S4** and **Supplementary File**.

### The morphological parameter and BMD

We also detected a total of 13 pairs between morphological parameter (MP) traits and BMD (**Supplementary Figure S11**, *p*< 0.05), with as many as eight traits from the T-cell panel. Of these, SSC-A on CD14^+^ monocyte exhibited the most significant association with BMD (*p* = 7.61E−05). After FDR correction, five association pairs were still significant (**Supplementary Table S4**) (FDR-adjusted *p*< 0.05). With respect to these most significant pairs, no evidence of heterogeneity was presented by Cochran’s Q statistic test (**Supplementary Figure S12**), and consistent association signals were confirmed by using different MR methods (**Supplementary Figure S13**). The signal, SSC-A on monocyte (**Supplementary Figure S14**), was simultaneously associated with TB-, FN-, and LS-BMD, suggesting that increased intracellular complexity of monocytes was associated with the higher BMD.

### Mediation analysis of immune traits on BMD

Since the above results highlighted the four immune traits on monocytes (CD14 versus CD16 classification) and the monocyte as the precursor of osteoclast plays a very important role in bone metabolism, it is well known that these immune cells have a complex interaction pattern in mediating complex diseases. We attempted to test the potential effects of the four immune traits on mediating the associations between other immune traits (with nominal significance) and BMD. As we expected, multiple immune traits could indirectly affect BMD *via* CD40 on monocytes. The CD27 molecule on several B-cell subsets was the most frequently observed signature in immune traits, followed by the CD25 molecule expressed by B cells (**Supplementary Table S9**), with the estimated mediation effects accounting for total effects were significant and high (e.g., 81.8% for CD27 on IgD^−^ CD38 and CD40 on CD14^+^ CD16^−^ monocyte and BMD). Additionally, significant mediation effects were also detected for the traits from other cells (e.g., CD4^+^ T cells and Treg cells).

### Multivariate MR analyses exclude potential pleiotropic effects of significant traits

Multivariable MR analysis with IVs for all identified immune traits showed that there existed strong evidence of independent causal effects for several trait types, including MFI, RC, and AC on BMDs (**Supplementary Figure S15**). After adjusting for effects of other identified immune traits in TB-BMD, the significant traits include CD45RA on CD39^+^ resting Treg (**Supplementary Figure S15A**), CD25 on resting Treg (**Supplementary Figure S15A**), CD33^br^ HLA DR^+^ CD14^−^ AC (**Supplementary Figure S15B**), CD39^+^ CD8^br^ AC (**Supplementary Figure S15B**), resting Treg %CD4, and %CD4 Treg (**Supplementary Figure S15C**), activated and resting Treg %CD4^+^ (**Supplementary Figure S15C**), and activated Treg %CD4 Treg (**Supplementary Figure S15C**). The independent effects of CD20^-^ CD38^−^ AC (**Supplementary Figure S15F**) on LS-BMD, CD40 on CD14^+^ CD16^−^ monocyte (**Supplementary Figure S15H**), and CD24 on memory B cell (**Supplementary Figure S15H**) on FA-BMD were validated. However, after the adjustment of some traits belonging to MP traits, the associations with LS-BMD became insignificant (**Supplementary Figure S15G**).

### The detected associations were supported by known interaction effects between immune cells and bone from previous studies

By searching the literature, we found that some of the detected causal associations between immune cells and the bone were supported by known interaction effects between immune cells and bone from previous studies (**Table 2**). Partially, the definite evidence has supported that CD40/CD40L signaling affects bone metabolism (36, 37). On the other hand, most of the detected associations were not supported by known functional evidence and were awaiting further functional studies.

**Table 2:** The known interaction effects between immune cells and bone metabolism from previous studies.

Traits	Factors	Biological effects	PMID
CD40 on monocytes	CD40/CD40L system	T-cell-deficient nude mice, CD40 KO mice, and CD40L KO mice displayed bone loss, increased bone resorption and diminished BM OPG production	17202317**†**
	CD40L	Activated CD40L^−/−^ CD4^+^ T cells expressing RANKL but deficient in INF-γ contribute to the profound generalized osteopenia found in patients with XHIM syndrome	17360404**†**
	CD40/CD40L system	The degree of methylation of CpGs in the CD40 promoter could contribute to the acquisition of BMD, whether the degree of methylation of the CD40 gene affects the level of CD40 expression is unclear	26545336**†**
	CD40L	Neither anti-CD40L mAb nor soluble CD40L altered OC formation in BMM and T-cell cultures stimulated with RANKL and M-CSF	18680714
	CD40/CD40L system	Neutralization of CD40L increased bone formation in vertebral bone by promoting Wnt-10b production	29522194
	CD40/CD40L system	CD40L^−/−^ mice was protected against bone loss, T cells regulate SC osteoclastogenic activity through CD40L, OVX increases TNF production of T cells through CD40L	21187391
	CD40L	CD40L accelerates osteoclastogenesis in the presence of RANKL and LPS	21521224
EM CD4^+^ and CM CD4^+^ T cell	–	A significant decrease in memory CD4^+^ (CD4^+^/CD27^+^/CD45RA^−^) T cells was observed in OP women	19876583
	TNF-α, IL-17A	Production of proinflammatory cytokines due to activation of memory T cells is required for acute phase bone loss leading to osteoporosis	31995253
	IL-4	MLN CD44^hi^CD62L^lo^CD4^+^T cells could promote bone damage *via* osteoclasts after migrating predominantly to the BM in mice fed with an egg-white diet	34326478
CD45RA on CD39^+^ resting Treg	CD45RA	Low CD39^+^/CD45RA^+^ Treg cells that may indicate loss of suppressive function	25421756**†**
	IL-10 and TGF-β	Delayed patients after bone fractures presented significantly higher resting Treg proportion	30946855**†**
Activated Treg %CD4 Treg	GM-CSF, IFN-γ, IL-5, and IL-10	Activated CD4^+^ CD25^+^ Treg cells regulate cytokine production and inhibit osteoclastogenesis *in vitro* and *in vivo*	18480308**†**
CD24 on memory B cell	GM-CSF	There were positive correlations between several altered subtypes B lymphocytes and BMD in women with osteoporosis	19876583

XHIM, X-linked hyper-IgM; BMD, bone mineral density; OVX, ovariectomy; KO, knockout; BM, bone marrow; OPG, osteoprotegerin; EM, effector memory; CM, central memory; SC, stroma cell; CD40L, ligand of CD40; RANKL, receptor activator of nuclear factor-kappaB ligand; M-CSF, macrophage colony-stimulating factor; INF-γ, interferon-γ; TNF-α, tumor necrosis factor-α; IL, interleukin; TGF-β, transforming growth factor-β; GM-CSF, granulocyte macrophage colony-stimulating factor; MLN, mesenteric lymph nodes; LPS, lipopolysaccharide.

†The reported results are consistent with the direction obtained from our analysis.

## Discussion

Although the immune system plays an important role in bone metabolism, its role in the progression of osteoporosis remains complex and unclear. To the best of our knowledge, this is the first MR analysis to explore the potential causal relationship of multiple types of immune traits with BMD. This study found 9, 16, 22, and 5 significant interaction pairs between four types of immune traits (i.e., MFI, RC, AC, and MP) and BMD, respectively. The monocyte subsets marked by a level of CD40 molecule were highlighted, as they presented top significant and consistent effects on BMD, and they had significant mediation effects to bridge the associations between other immune traits and BMD. This study also found that significant immune traits existed in different types of immune cells (mainly in monocyte, several subsets of T cell and B cells). Taken together, these findings described more comprehensive involvements of immune factors in bone metabolism than we expected.

Emerging evidence has suggested that the CD40/CD40L system is essential for T-cell activation and several functions of the immune system (e.g., activation and differentiation of macrophage) and involvement in bone metabolism (38). As shown in **Table 2**, the CD40 KO and CD40L KO mice and T-cell-deficient nude mice displayed more bone loss, increased bone resorption, and diminished OPG production (39). Moreover, previous findings from the human population have also demonstrated that low bone density is a frequent clinical feature in children with X-linked hyper-IgM syndrome, an inherited disorder caused by mutations in the gene encoding CD40L (40). The activated CD40L^−/−^ T cells from both humans and mice promote robust osteoclast differentiation of monocyte due to the deficiency in IFN-γ production (40). These studies have shown that the CD40L/CD40 system is required to maintain normal bone modeling and remodeling.

On the contrary, the mouse model lacking CD40L in T cells is resistant to bone loss induced by ovariectomy (OVX) (41). CD40L could accelerate osteoclastogenesis in the presence of RANKL and lipopolysaccharide (LPS) (36). However, neither anti-CD40L mAb nor soluble CD40L can alter osteoclast formation in bone marrow monocyte (BMM), and T-cell cultures can stimulate osteoclast with RANKL and M-CSF (42). In addition, another animal study has reported that neutralization of CD40L can increase bone formation in vertebral bone by promoting Wnt-10b production (43). A genetic and functional study (37) has revealed that the degree of methylation of CpGs in the CD40 promoter contributes to the acquisition of BMD and that downregulation of OPG levels can be responsible for the lower BMD observed in TT women for rs1883832 of the CD40 gene. Furthermore, a recent study shows that rs1883832 T allele is indeed associated with decreased CD40 expression and increased CD27 expression on B-cell subsets (24). Although many open questions remain, discoveries covering T-cell-derived CD40L signals to B cells, through the CD40/CD40L system, have demonstrated some mechanisms underlying the correlation between controlling bone homeostasis and the immune response.

Currently, one probably acceptable explanation for the association between the CD40/CD40L system and bone metabolism is that chronic deficiency of CD40L costimulatory molecules (as in CD40L knockout mouse models) may lead to impaired immune system development and dysfunctional adaptive immune responses, which easily results in producing large numbers of defective B cells characterized by reduced OPG production. In contrast, in individuals with a mature immune system, suppression of CD40L may particularly complicate bone regulation mediated by crosstalk between T cells and other various cells. The known mechanisms are far from fully explained for the associations between bone and CD40 on monocyte, and further functional studies are still needed to completely disclose their relationship. Our findings may provide some clues for an explanation of the dual mechanism for the CD40/CD40L system involved in osteoporosis development.

Focusing on MFI or cell count traits of lymphocytes, our MR results also supported significant causal effects of several cell populations on the bone. Previous studies have reported that in B- and T-cell-deficient mice, these lymphocytes affect bone homeostasis. The production of proinflammatory cytokines due to activation of memory T cells can cause acute phase bone loss and even osteoporosis. A study has hypothesized that there are more TNF-α and IL‐17A producing memory T cells in postmenopausal women with osteoporosis (44). EM CD4^+^ T cells in mesenteric lymph nodes mediate bone loss in food-allergic enteropathy model mice by creating IL-4 dominance (45). These studies are somewhat inconsistent with our reported results that memory T cells (e.g., EM CD4^+^ AC, CM CD4^+^ %CD4^+^, and EM CD4^+^ %T cell) may be protective against bone loss. However, one population study found that memory CD4^+^ T cells were positively associated with FN-BMD (18), providing some evidence for the reliability of our results. Currently, the interactions exist between T cells and bone metabolism, but the fully underlying mechanisms are complex and largely unknown.

In our results, Treg cells also have interaction effects on bone phenotypes. For example, increased CD45RA expression on CD39^+^ resting Treg, increased expression of CD25 on resting Treg, and higher level of CD24 on memory B cell may be implicated in predisposition to osteoporosis. The low expression of CD45RA within the Treg population probably has a limited ability to upregulate inhibitory cytokines, leading to loss of suppressive function (46, 47). Recent findings showed that delayed union patients with isolated closed tibial fracture presented a significantly lower percentage of EM (CD45RA^−^ CD62L^−^) Treg population and significantly higher naive Treg (CD45RA^+^ CD62L^+^) proportion when compared to normal patients. Among the naive, EM, and CM Treg cells, the EM Treg cells were considered the most potential cells for suppressing RANKL expression in T conventional cells (47). Kelchtermans et al. (48) reported the close interconnection between activated Treg and bone cells, as evidenced by the pre-stimulated Treg cells inhibiting osteoclastogenesis by increasing the expression of cytokines such as GM-CSF and IFN-γ *in vivo* and *in vitro*. Collectively, these observations are in line with our findings, indicating that an increased level of CD45RA on naive Treg is associated with a predisposition to osteoporosis, while the higher activated and secreting Treg proportion is correlated with protection against osteoporosis.

Furthermore, our MR results highlighted the contribution of CD39-positive cell (e.g., CD39^+^ CD4^+^ %CD4^+^, CD39^+^ CD8^br^ %T cell, and CD39^+^ resting Treg %CD4 Treg) in exacerbating the progress of osteoporosis. As previously described (24), the majority of cell populations with high heritability (60%) were positive for CD39 markers. CD39 is a transmembrane hydrolase that degrades extracellular ATP to adenosine, and it has an anti-inflammatory function by reducing extracellular pro-inflammatory ATP. It is also involved in the suppressive function of a variety of immune cells (e.g., Foxp3^+^ Tregs, CD8^+^ Tregs) (49), and the function between adenosine and bone remodeling is particularly complicated by the abundance of receptors (50). Even if we do not find relevant evidence, this may be used to explain the reverse causality between BMD and CD39^+^ CD4^+^ %T cell found in our MR analysis results. Meanwhile, the potential functional mechanism needs to be further verified.

In our study, some traits from B cells were significantly associated with bone phenotypes. During an inflammatory status such as rheumatoid arthritis and periodontitis, the memory B cells (e.g., CD27^+^CD38^−^ memory B cells) tend to improve RANKL expression and osteoclastogenesis and support osteoclast differentiation *in vitro* in a RANKL-dependent manner (51–54), which probably supported the findings from our mediation analyses that the potential associations of several memory B cells expressing CD25 or CD27 with BMD can be mediated by CD40 on monocytes. CD27 has recently been defined as a marker for memory B cells. Synergistic enhancement of immunoglobulin G (IgG) production by IL-10 and CD70 transfectants was evident in highly purified CD27^+^ B cells but not in CD27^−^ B cells (55). This molecule, IgG, seems to play an important role in regulating osteoclastogenesis: IgG immunocomplexes are generally considered an important pathway leading to bone destruction in rheumatoid arthritis by driving the differentiation of human blood monocytes into a nuclear factor of activated T-cell cytoplasmic 1 (NFATc1)-negative non-classical osteoclasts and RANKL-induced classical osteoclasts (56). So far, since memory B lymphocytes play a major role in the immune response to infection, the role of these cell populations marked by various immune molecules in postmenopausal osteoporosis has not been adequately described. A previous study reported a positive correlation between altered memory B lymphocytes and both FN- and LS-BMD in women with osteoporosis (18), which was inconsistent with our reported results of mediation analyses, in which there were negative causal relationships between the total effect of several memory B cells (e.g., early Bm5 and late Bm5 memory cell) with BMD and a negative effect of CD24 on memory B cell on BMD in MR analyses. Further functional studies are needed to confirm these observations.

Four types of immune traits (i.e., MFI, RC, AC, and MP) describe the basic characteristics from different directions. We observed the largest number of causal associations between RC levels and BMD when compared with the other three types of traits. The RC trait, as opposed to the AC trait, reflects not the absolute cell count of a particular immune cell but its proportion relative to the corresponding progenitor cell lineage. The relative ratio of immune cells probably truly reflects the balance between immune cells and immune factors, which will affect bone metabolism. As we reported, among the RC traits, several subpopulations representing Treg activation status (e.g., activated Treg/resting Treg/secreting Treg %CD4 Treg) and CD4 T-cell differentiation status (e.g., CM CD4^+^ %CD4^+^, EM CD4^+^ %T cell, and TD CD4^+^ %CD4^+^), with respect to the proportion of precursor cells, had significant causal associations with BMD in different patterns. Furthermore, during immune homeostasis, there is a balance between the activity of pro- and anti-inflammatory T cells, thereby maintaining immune surveillance while avoiding autoimmunity, whereas the differentiation of naive CD4 T cells can be controlled by several key immune molecules (57). We, therefore, proposed the hypothesis that, in the molecular and cellular crosstalk between the skeletal and immune system, these percentages may also reveal associations with changes in immune molecules responsible for factors involved in feedback mechanisms maintaining the balance between cells. Further functional studies are needed to explain the effects of changes in cell-type proportions on bone metabolism. By detecting and tracking the balance state between these cells, it is expected to provide new insights into the prevention of osteoporosis.

Although significant associations were detected for all four BMD outcomes (TB-, LS-, FA-, and FN-BMD), the number of significant pairs for TB-BMD was the largest. Some factors may explain this observation. First, the sample size used for TB-BMD was larger than for other BMD phenotypes. Second, previous studies have found that the distribution of immune cells has a difference between microanatomical regions in physiological conditions (53, 58). Cancellous bone is the first target site where immune cells contribute to the development of osteoporosis. Immune cells may also migrate from the bone marrow with the aid of capillaries to the depth of the cortical bone. For example, our results highlighted that CD40 on CD14^+^ CD16^−^ monocyte remained causally associated only with TB- and FA-BMD after FDR correction, suggesting the specificity of monocytes to the cortical bone predominant bone tissue, which is almost consistent with the reported migration pattern. Upon activation, monocytes migrate from the center to the perisinusoidal basement membrane, into the vascular sinusoids and venous capillaries, and through the Haversian and Walker tubes to the cortical bone, until the systemic circulation (59). In addition, it is known that the adult skeleton is largely composed of 80% cortical bone and 20% trabeculae, of which the vertebrae are composed of cortical bone and trabecular bone in a ratio of 25:75; the ratio of the femoral head is 50:50 and 95:5 in radial diaphysis (60). Therefore, we hypothesized that the different patterns and strength of immune cell action on the four sites of skeletal were due to the different components of bone tissue.

In conclusion, this study highlights the complex interaction patterns between the immune and bone systems. The underlying mechanisms for the detected causal associations still need to be elicited. The findings have extended the discoveries of osteoimmunology and disclosed immune skeletal interface, whose practical implications may provide helpful clues for the prevention and intervention of osteoporosis.

## Data availability statement

The original contributions presented in the study are included in the article/**Supplementary Material**. Further inquiries can be directed to the corresponding authors.

## Author contributions

S-FL, F-YD, R-RC, and X-HY conceived the design of the study. X-HY and X-TL obtained the data. M-FX and X-HY cleared up the datasets. R-RC and X-HY mainly performed the data analyses. S-FL, F-YD, R-RC, X-HY, and M-FX drafted and revised the manuscript. All authors contributed to the article and approved the submitted version.

## Funding

The study was supported by Natural Science Foundation of China (81872681 8217120656, 8217120657, and 8210120875), the Science and Technology Project of Suzhou (SS202050 and SYS2019024), the QingLan Research Project of Jiangsu Province, and a Project of the Priority Academic Program Development of Jiangsu Higher Education Institutions.

## Acknowledgments

We thank all the GEFOS consortium studies for making the summary association statistics data publicly available, and we are grateful of all the investigators and participants in a longitudinal study (SardiNIA project) for SardiNIA dataset and their contribution to those studies.

## Conflict of interest

The authors declare that the research was conducted in the absence of any commercial or financial relationships that could be construed as a potential conflict of interest.

## Publisher’s note

All claims expressed in this article are solely those of the authors and do not necessarily represent those of their affiliated organizations, or those of the publisher, the editors and the reviewers. Any product that may be evaluated in this article, or claim that may be made by its manufacturer, is not guaranteed or endorsed by the publisher.
