# Supplementary material for: The immune factors have complex causal regulation effects on bone mineral density
Source: Front Immunol. 2022 Oct 20;13:959417. doi: 10.3389/fimmu.2022.959417 (PMC9630477; doi:10.3389/fimmu.2022.959417)
Supplement: Supplementary file 1 [file DataSheet_1.zip › Data Sheet 1/959417-Supplementary-Material/FiguresS1-S15.docx]

**
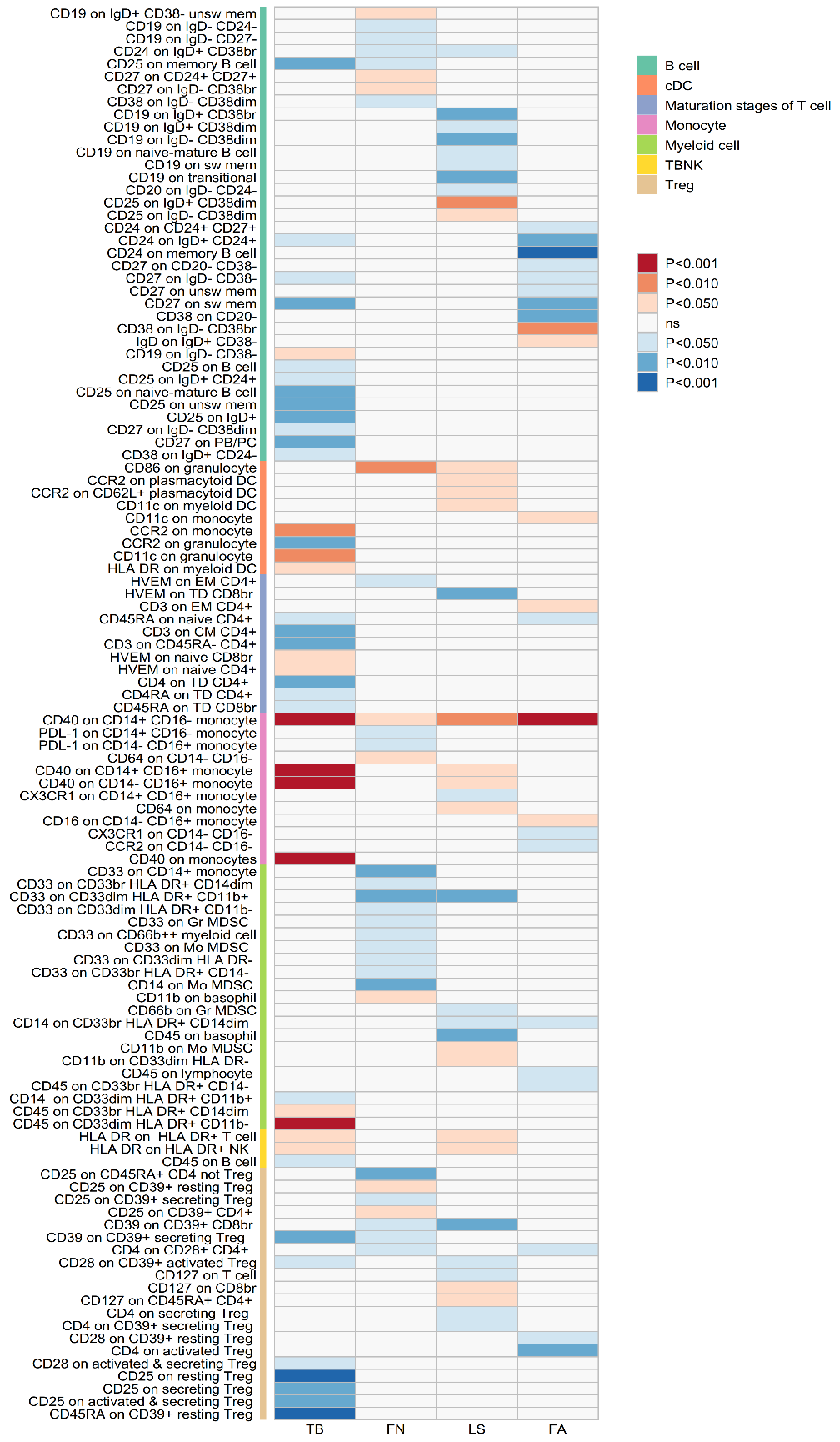
**

Figure S1. Mendelian randomization associations of immune traits (MFI) on BMDs that derived from the IVW analysis. MFIs, median fluorescence intensities; IVW, inverse-variance weighted; BMD, bone mass density.


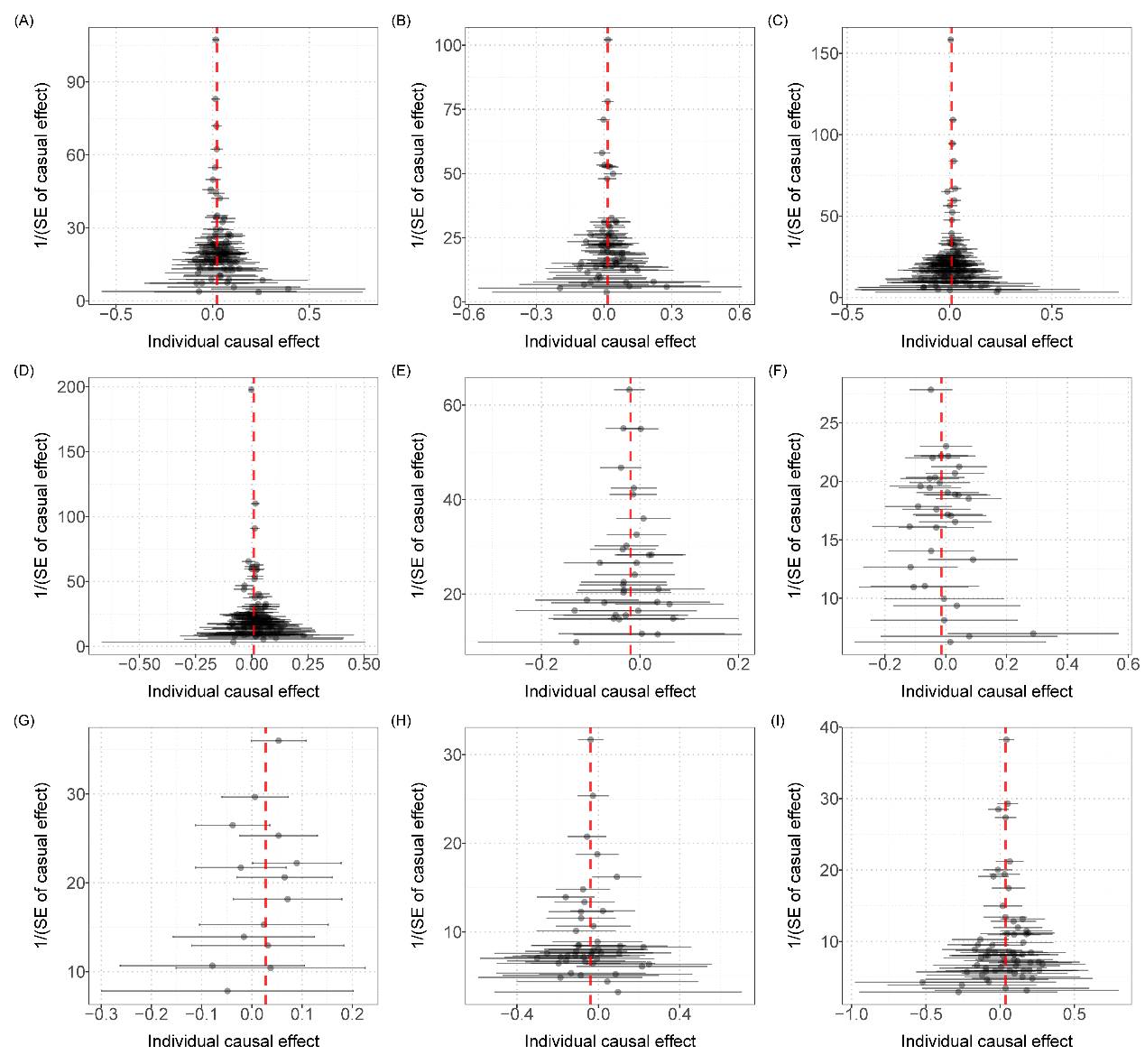


Figure S2. Funnel plot for the relationship between the SNP effect size of causal immune traits (MFI) and the corresponding effect size estimates of BMDs. (A) CD40 on CD14^+^ CD16^+^ monocyte on TB-BMD. (B) CD40 on CD14^+^ CD16^-^ monocyte on TB-BMD, (C) CD40 on CD14^-^ CD16^+^ monocyte on TB-BMD, (D) CD40 on monocytes on TB-BMD, (E) CD45RA on CD39^+^ resting Treg on TB-BMD, (F) CD25 on resting Treg on TB-BMD, (G) CD45 on CD33^dim^ HLA DR^+^ CD11b^-^ on TB-BMD, (H) CD24 on memory B cell on FA-BMD, (I) CD40 on CD14^+^ CD16^-^ monocyte on FA-BMD. MFIs, median fluorescence intensities; TB, total body; FA, forearm; BMD, bone mineral density.


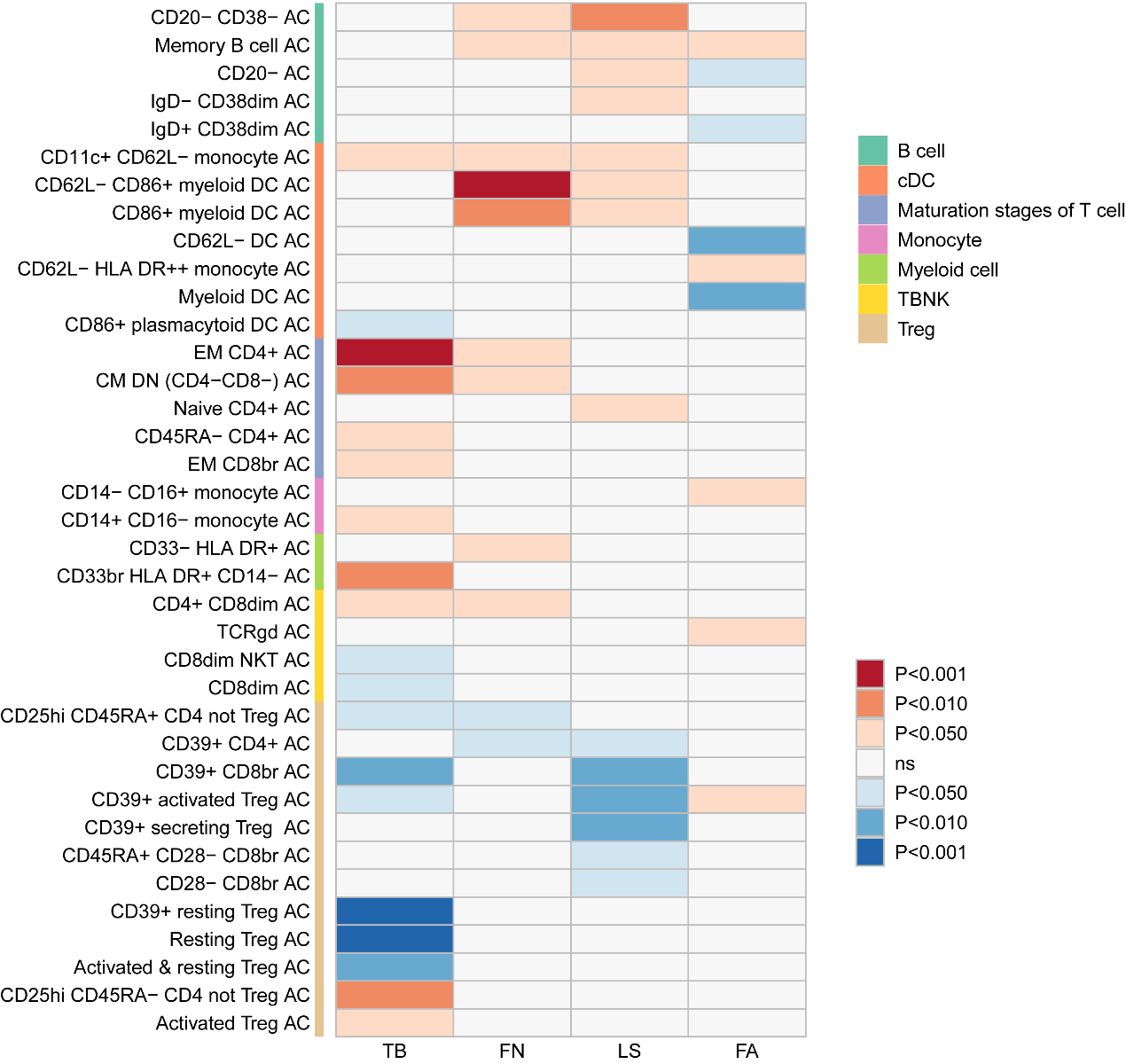


Figure S3. Mendelian randomization associations of immune traits (AC) on BMDs that derived from the IVW analysis. AC, absolute count; IVW, inverse-variance weighted; BMD, bone mass density.


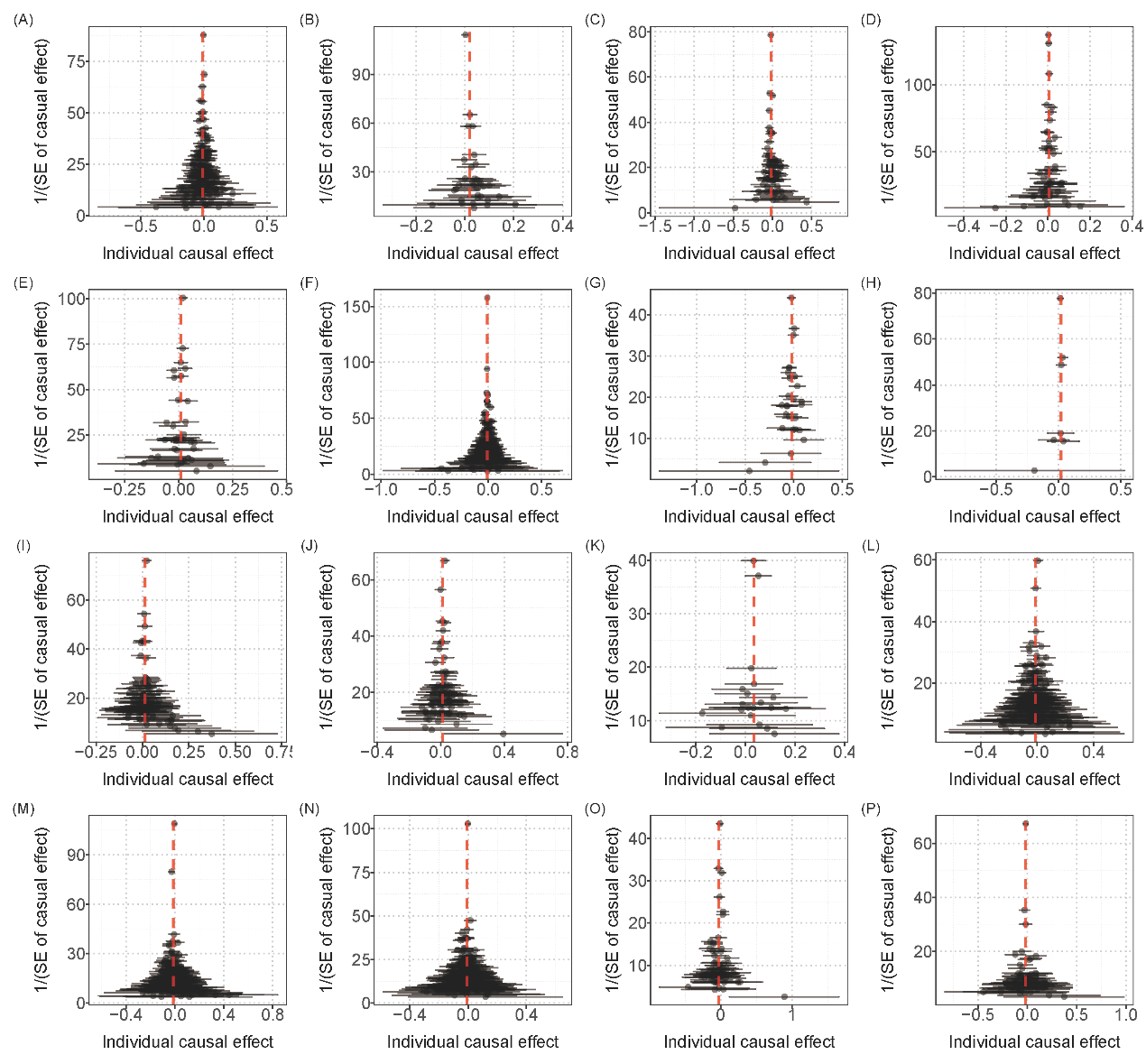


Figure S4. Funnel plot for the relationship between the SNP effect size of causal immune traits (AC) and the corresponding effect size estimates of BMDs. (A) CD39^+^ resting Treg AC on TB-BMD, (B) EM CD4^+^ AC on TB-BMD, (C) Resting Treg AC on TB-BMD, (D) CD33br HLA DR^+^ CD14^-^ AC on TB-BMD, (E) CD25^hi^ CD45RA^-^ CD4 not Treg AC on TB-BMD, (F) CD39^+^ CD8^br^ AC on TB-BMD, (G) Activated & resting Treg AC on TB-BMD, (H) CM DN (CD4^-^CD8^-^) AC on TB-BMD, (I) CD62L^-^ CD86^+^ myeloid DC AC on FN-BMD, (J) CD86^+^ myeloid DC AC on FN-BMD, (K) CD20^-^ CD38^-^ AC on LS-BMD, (L) CD39^+^ activated Treg AC on LS-BMD, (M) CD39^+^ secreting Treg AC on LS-BMD, (N) CD39^+^ CD8^br^ AC on LS-BMD, (O) Myeloid DC AC on FA-BMD, (P) CD62L- DC AC on FA-BMD. AC, absolute count; EM, effector memory; CM, central memory; TB, total body; FN, femoral neck; LS, lumbar spine; FA, forearm; BMD, bone mineral density.


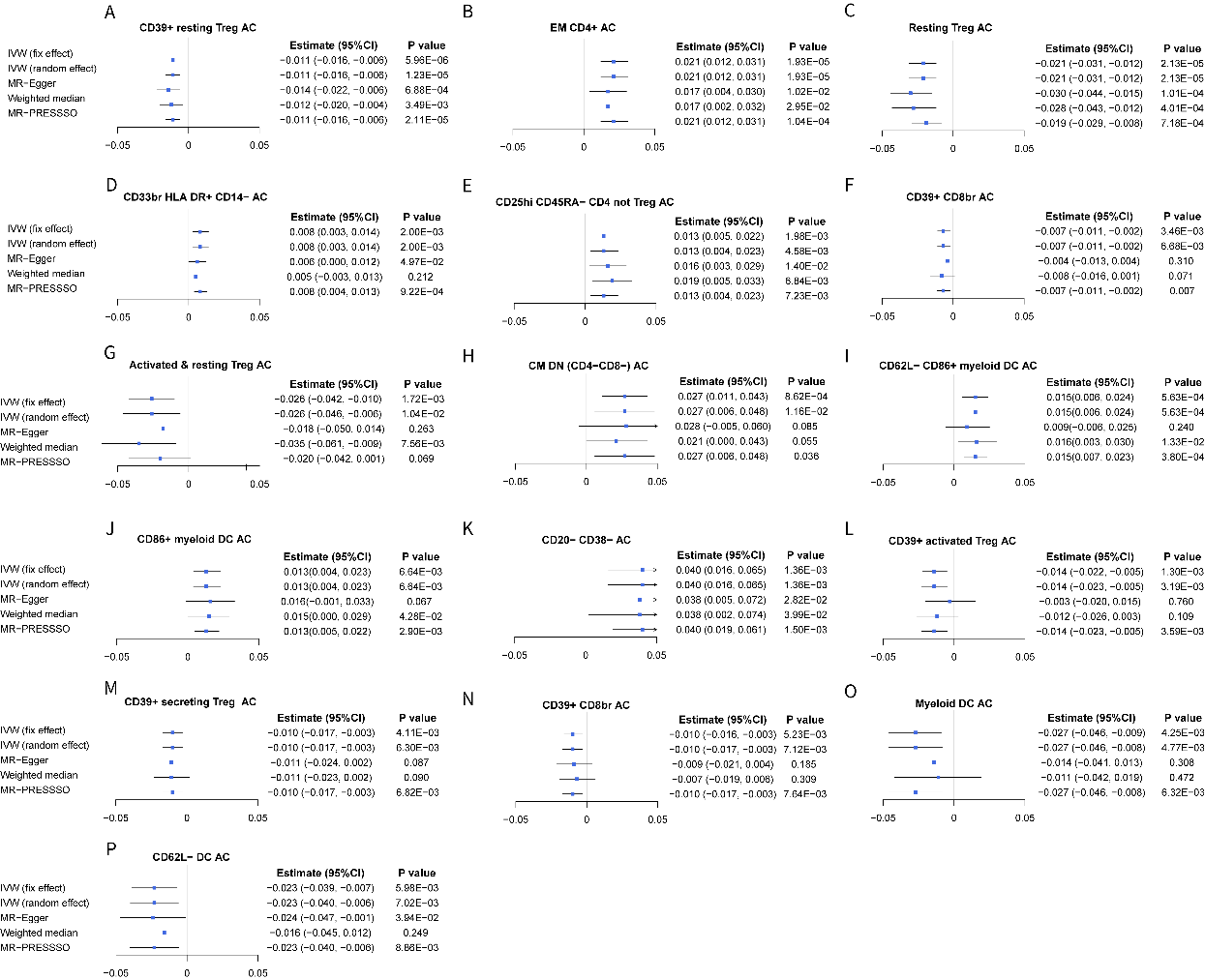


Figure S5. Forest plot of causal effects for different immune traits (AC) on BMDs. (A) CD39^+^ resting Treg AC on TB-BMD, (B) EM CD4^+^ AC on TB-BMD, (C) Resting Treg AC on TB-BMD, (D) CD33br HLA DR^+^ CD14^-^ AC on TB-BMD, (E) CD25^hi^ CD45RA^-^ CD4 not Treg AC on TB-BMD, (F) CD39^+^ CD8^br^ AC on TB-BMD, (G) Activated & resting Treg AC on TB-BMD, (H) CM DN (CD4^-^CD8^-^) AC on TB-BMD, (I) CD62L^-^ CD86^+^ myeloid DC AC on FN-BMD, (J) CD86^+^ myeloid DC AC on FN-BMD, (K) CD20^-^ CD38^-^ AC on LS-BMD, (L) CD39^+^ activated Treg AC on LS-BMD, (M) CD39^+^ secreting Treg AC on LS-BMD, (N) CD39^+^ CD8^br^ AC on LS-BMD, (O) Myeloid DC AC on FA-BMD, (P) CD62L^-^ DC AC on FA-BMD. AC, absolute count; EM, effector memory; CM, central memory; TB, total body; FN, femoral neck; LS, lumbar spine; FA, forearm; BMD, bone mineral density.


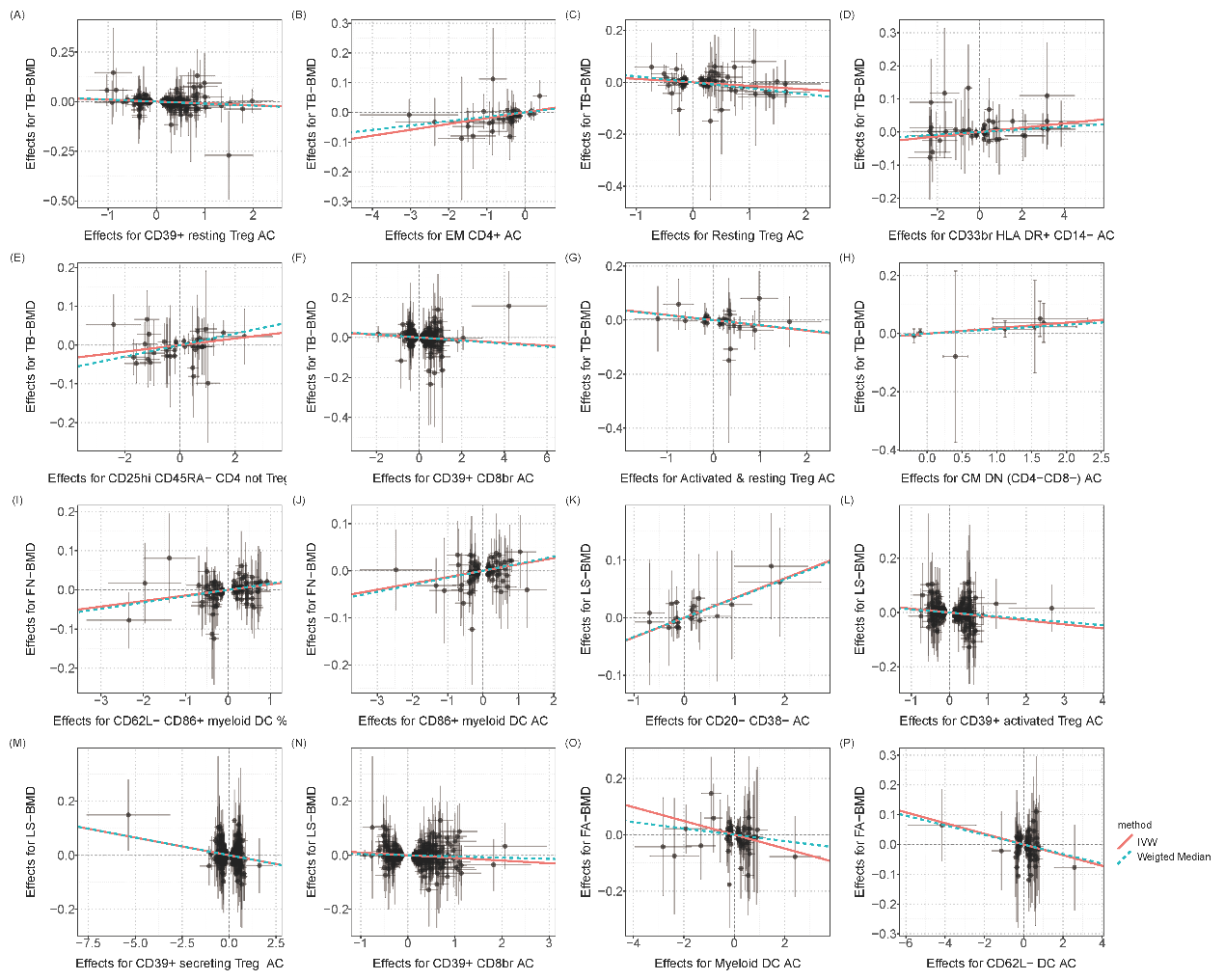


Figure S6. Scatter plot for the relationship between the SNP effect size of causal immune traits (x-axis) and the corresponding effect size estimates of BMDs (y-axis). (A) CD39^+^ resting Treg AC on TB-BMD. (B) EM CD4^+^ AC on TB-BMD, (C) Resting Treg AC on TB-BMD, (D) CD33br HLA DR^+^ CD14^-^ AC on TB-BMD, (E) CD25^hi^ CD45RA^-^ CD4 not Treg AC on TB-BMD, (F) CD39^+^ CD8^br^ AC on TB-BMD, (G) Activated & resting Treg AC on TB-BMD, (H) CM DN (CD4^-^CD8^-^) AC on TB-BMD, (I) CD62L^-^ CD86^+^ myeloid DC AC on FN-BMD, (J) CD86^+^ myeloid DC AC on FN-BMD, (K) CD20^-^ CD38^-^ AC on LS-BMD, (L) CD39^+^ activated Treg AC on LS-BMD, (M) CD39^+^ secreting Treg AC on LS-BMD, (N) CD39^+^ CD8^br^ AC on LS-BMD, (O) Myeloid DC AC on FA-BMD, (P) CD62L^-^ DC AC on FA-BMD. AC, absolute count; EM, effector memory; CM, central memory; TB, total body; FN, femoral neck; LS, lumbar spine; FA, forearm; BMD, bone mineral density.


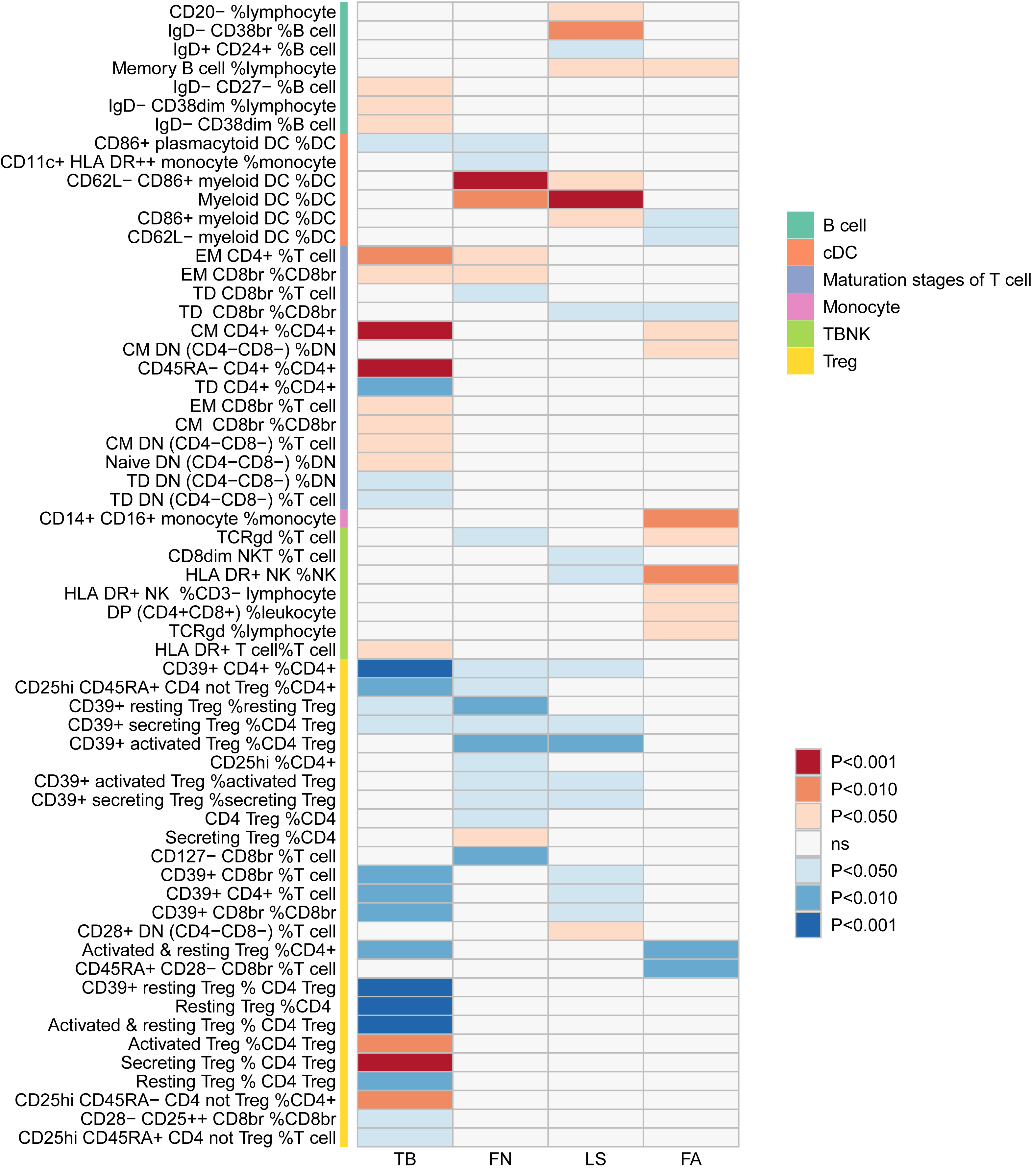


Figure S7. Mendelian randomization associations of immune traits (RC) on BMDs that derived from the IVW analysis. RC, relative count; IVW, inverse-variance weighted; BMD, bone mass density.


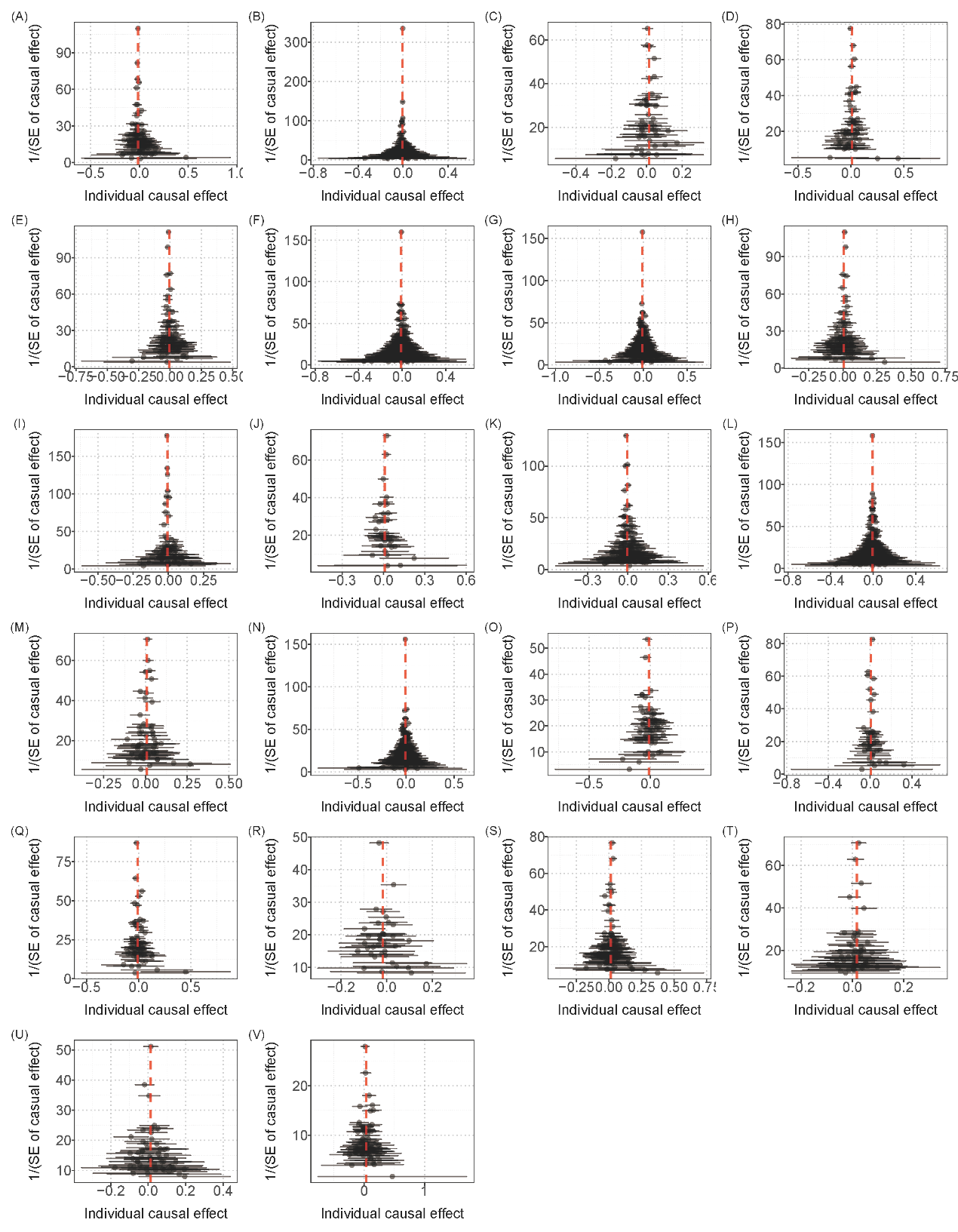


Figure S8. Funnel plot for the relationship between the SNP effect size of causal immune traits (RC) and the corresponding effect size estimates of BMDs. (A) Resting Treg %CD4 on TB-BMD. (B) CD39^+^ resting Treg %CD4 Treg on TB-BMD, (C) CM CD4^+^ %CD4^+^ on TB-BMD, (D) CD45RA^-^ CD4^+^ %CD4^+^ on TB-BMD, (E) Activated & resting Treg %CD4 Treg on TB-BMD, (F) CD39^+^ CD4^+^ %CD4^+^ on TB-BMD, (G) CD39^+^ CD8^br^ %T cell on TB-BMD, (H) Secreting Treg %CD4 Treg on TB-BMD, (I) CD25^hi^ CD45RA^+^ CD4 not Treg %CD4^+^ on TB-BMD, (J) Activated Treg %CD4 Treg on TB-BMD, (K) Resting Treg %CD4 Treg on TB-BMD, (L) CD39^+^ CD4^+^ %T cell on TB-BMD, (M) EM CD4^+^ %T cell on TB-BMD, (N) CD39^+^ CD8^br^ %CD8^br^ on TB-BMD, (O) TD CD4^+^ %CD4^+^ on TB-BMD, (P) CD25^hi^ CD45RA^-^ CD4 not Treg %CD4^+^ on TB-BMD, (Q) Activated & resting Treg %CD4^+^ on TB-BMD, (R) CD28^-^ CD25^++^ CD8^br^ %CD8^br^ on TB-BMD, (S) CD62L^-^ CD86^+^ myeloid DC %DC on FN-BMD, (T) Myeloid DC %DC on FN-BMD, (U) Myeloid DC %DC on LS-BMD, (V) CD14^+^ CD16^+^ monocyte %monocyte and FA-BMD. RC, relative count; EM, effector memory; CM, central memory; TD, terminally differentiated; TB, total body; FN, femoral neck; LS, lumbar spine; FA, forearm; BMD, bone mineral density.


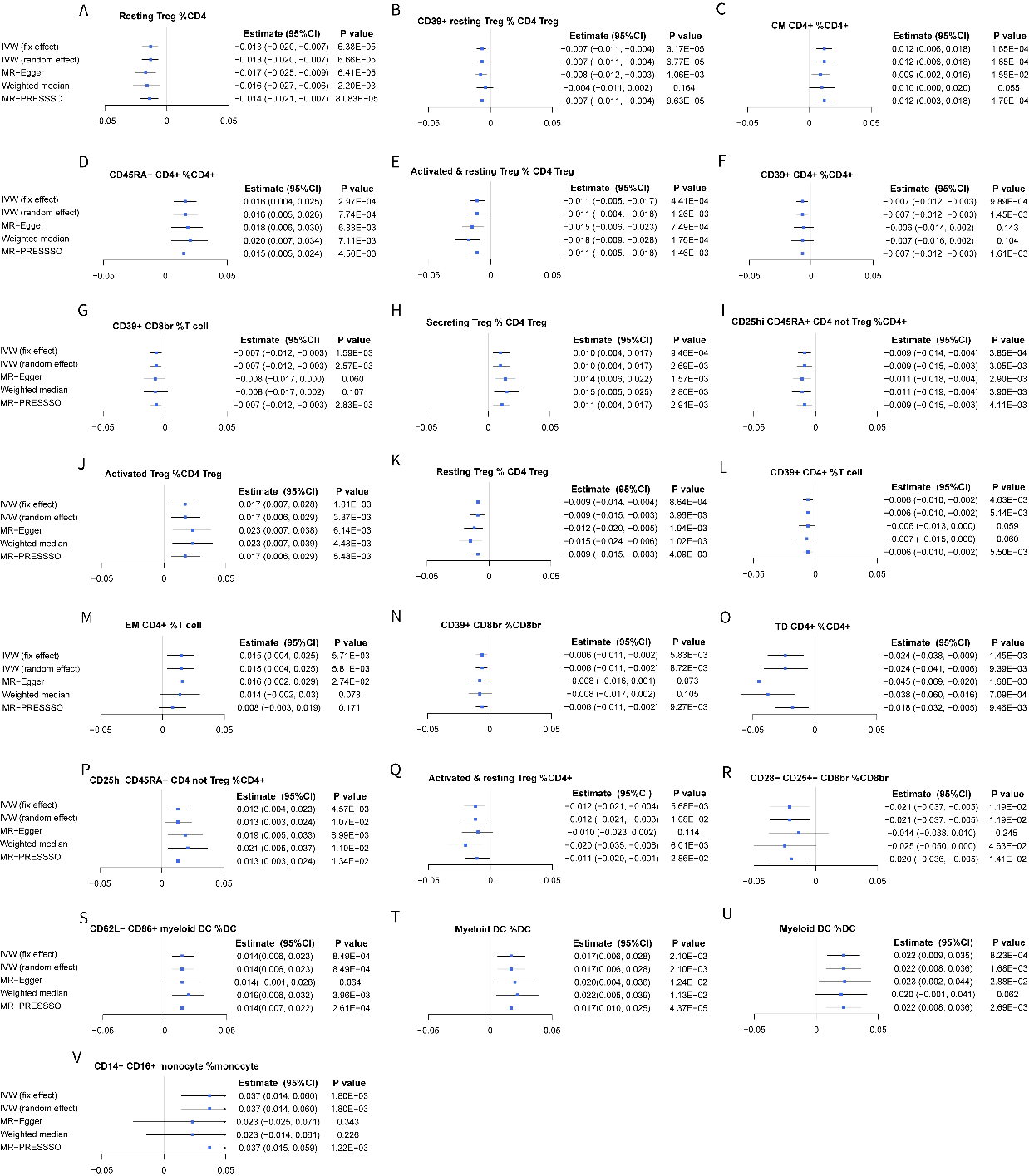


Figure S9. Forest plot of causal effects for different immune traits (RC) on BMDs. (A) Resting Treg %CD4 on TB-BMD. (B) CD39^+^ resting Treg %CD4 Treg on TB-BMD, (C) CM CD4^+^ %CD4^+^ on TB-BMD, (D) CD45RA^-^ CD4^+^ %CD4^+^ on TB-BMD, (E) Activated & resting Treg %CD4 Treg on TB-BMD, (F) CD39^+^ CD4^+^ %CD4^+^ on TB-BMD, (G) CD39^+^ CD8^br^ %T cell on TB-BMD, (H) Secreting Treg %CD4 Treg on TB-BMD, (I) CD25^hi^ CD45RA^+^ CD4 not Treg %CD4^+^ on TB-BMD, (J) Activated Treg %CD4 Treg on TB-BMD, (K) Resting Treg %CD4 Treg on TB-BMD, (L) CD39^+^ CD4^+^ %T cell on TB-BMD, (M) EM CD4^+^ %T cell on TB-BMD, (N) CD39^+^ CD8^br^ %CD8^br^ on TB-BMD, (O) TD CD4^+^ %CD4^+^ on TB-BMD, (P) CD25^hi^ CD45RA^-^ CD4 not Treg %CD4^+^ on TB-BMD, (Q) Activated & resting Treg %CD4^+^ on TB-BMD, (R) CD28^-^ CD25^++^ CD8^br^ %CD8^br^ on TB-BMD, (S) CD62L^-^ CD86^+^ myeloid DC %DC on FN-BMD, (T) Myeloid DC %DC on FN-BMD, (U) Myeloid DC %DC on LS-BMD, (V) CD14^+^ CD16^+^ monocyte %monocyte and FA-BMD. RC, relative count; EM, effector memory; CM, central memory; TD, terminally differentiated; TB, total body; FN, femoral neck; LS, lumbar spine; FA, forearm; BMD, bone mineral density.


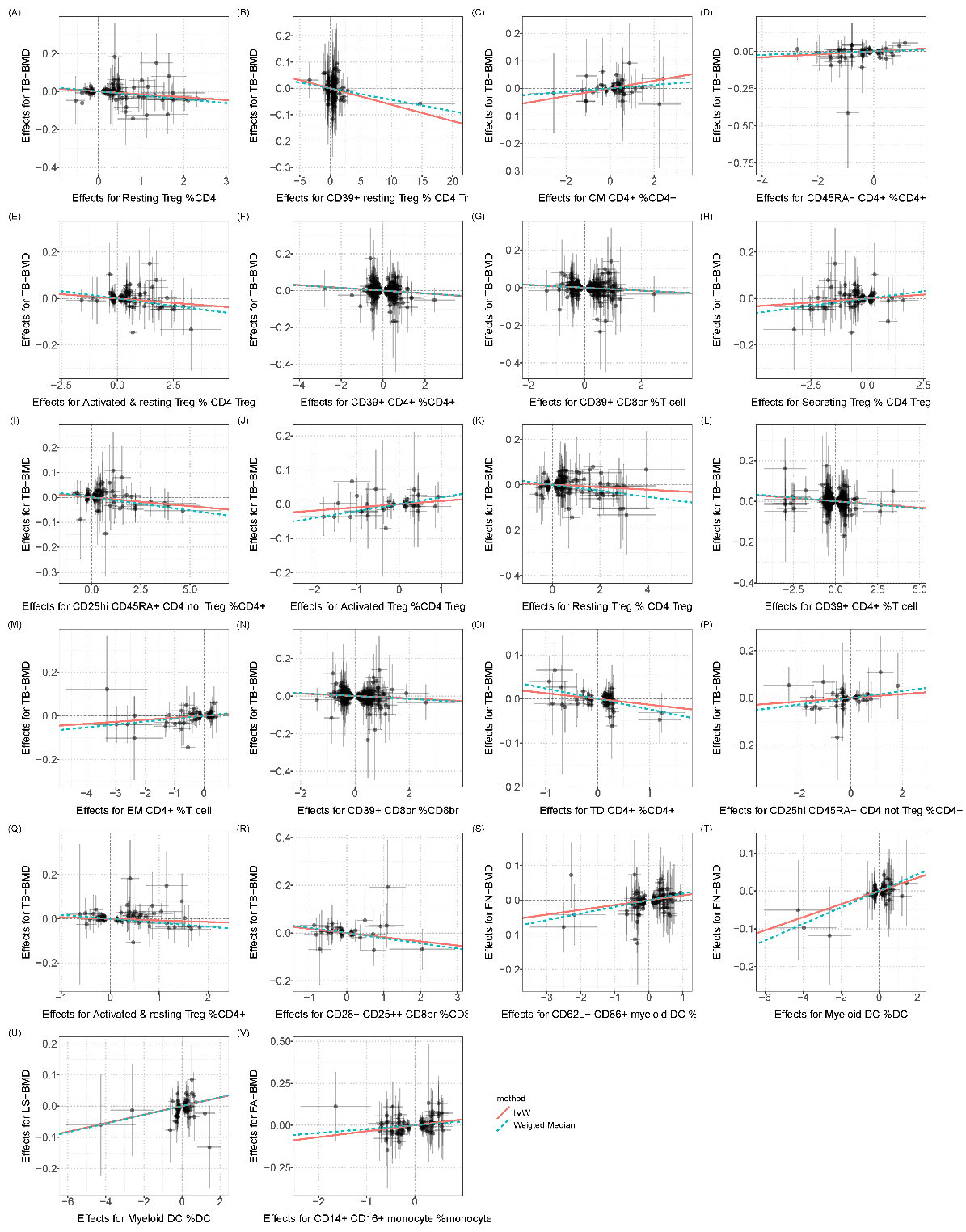


Figure S10. Scatter plot for the relationship between the SNP effect size of causal immune traits (x-axis) and the corresponding effect size estimates of BMDs (y-axis). (A) Resting Treg %CD4 on TB-BMD. (B) CD39^+^ resting Treg %CD4 Treg on TB-BMD, (C) CM CD4^+^ %CD4^+^ on TB-BMD, (D) CD45RA^-^ CD4^+^ %CD4^+^ on TB-BMD, (E) Activated & resting Treg %CD4 Treg on TB-BMD, (F) CD39^+^ CD4^+^ %CD4^+^ on TB-BMD, (G) CD39^+^ CD8^br^ %T cell on TB-BMD, (H) Secreting Treg %CD4 Treg on TB-BMD, (I) CD25^hi^ CD45RA^+^ CD4 not Treg %CD4^+^ on TB-BMD, (J) Activated Treg %CD4 Treg on TB-BMD, (K) Resting Treg %CD4 Treg on TB-BMD, (L) CD39^+^ CD4^+^ %T cell on TB-BMD, (M) EM CD4^+^ %T cell on TB-BMD, (N) CD39^+^ CD8^br^ %CD8^br^ on TB-BMD, (O) TD CD4^+^ %CD4^+^ on TB-BMD, (P) CD25^hi^ CD45RA^-^ CD4 not Treg %CD4^+^ on TB-BMD, (Q) Activated & resting Treg %CD4^+^ on TB-BMD, (R) CD28^-^ CD25^++^ CD8^br^ %CD8^br^ on TB-BMD, (S) CD62L^-^ CD86^+^ myeloid DC %DC on FN-BMD, (T) Myeloid DC %DC on FN-BMD, (U) Myeloid DC %DC on LS-BMD, (V) CD14^+^ CD16^+^ monocyte %monocyte and FA-BMD. RC, relative count; EM, effector memory; CM, central memory; TD, terminally differentiated; TB, total body; FN, femoral neck; LS, lumbar spine; FA, forearm; BMD, bone mineral density.


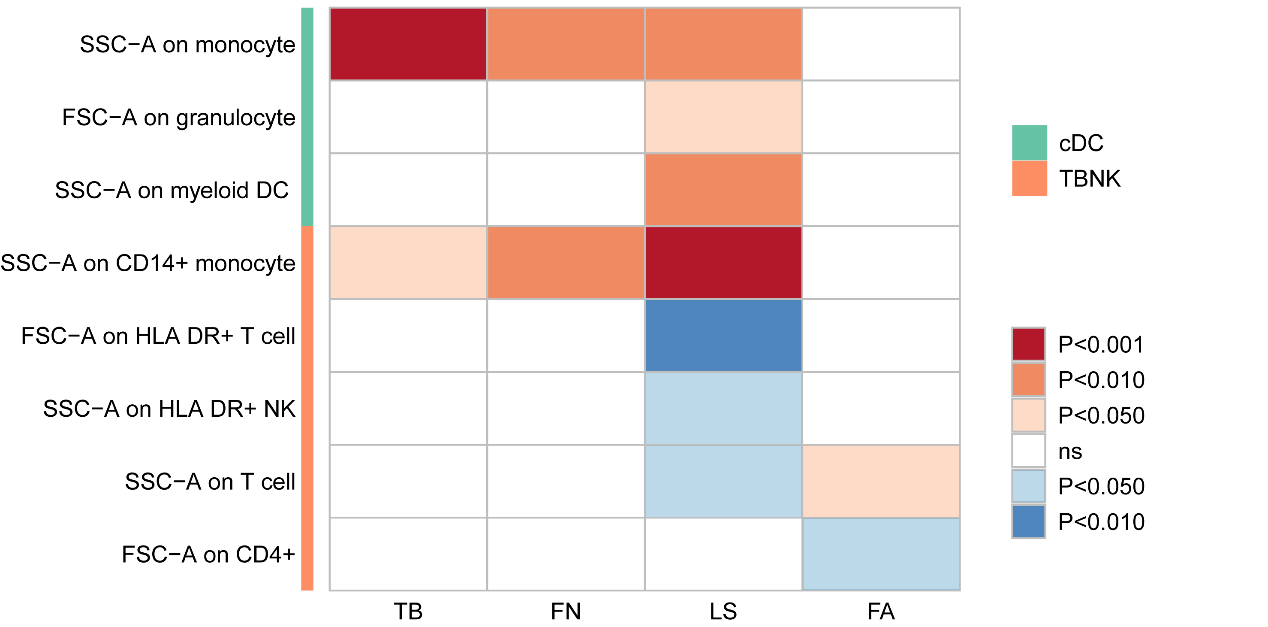


Figure S11. Mendelian randomization associations of immune traits (MP) on BMDs that derived from the IVW analysis. MP, morphological parameter; IVW, inverse-variance weighted; BMD, bone mass density.


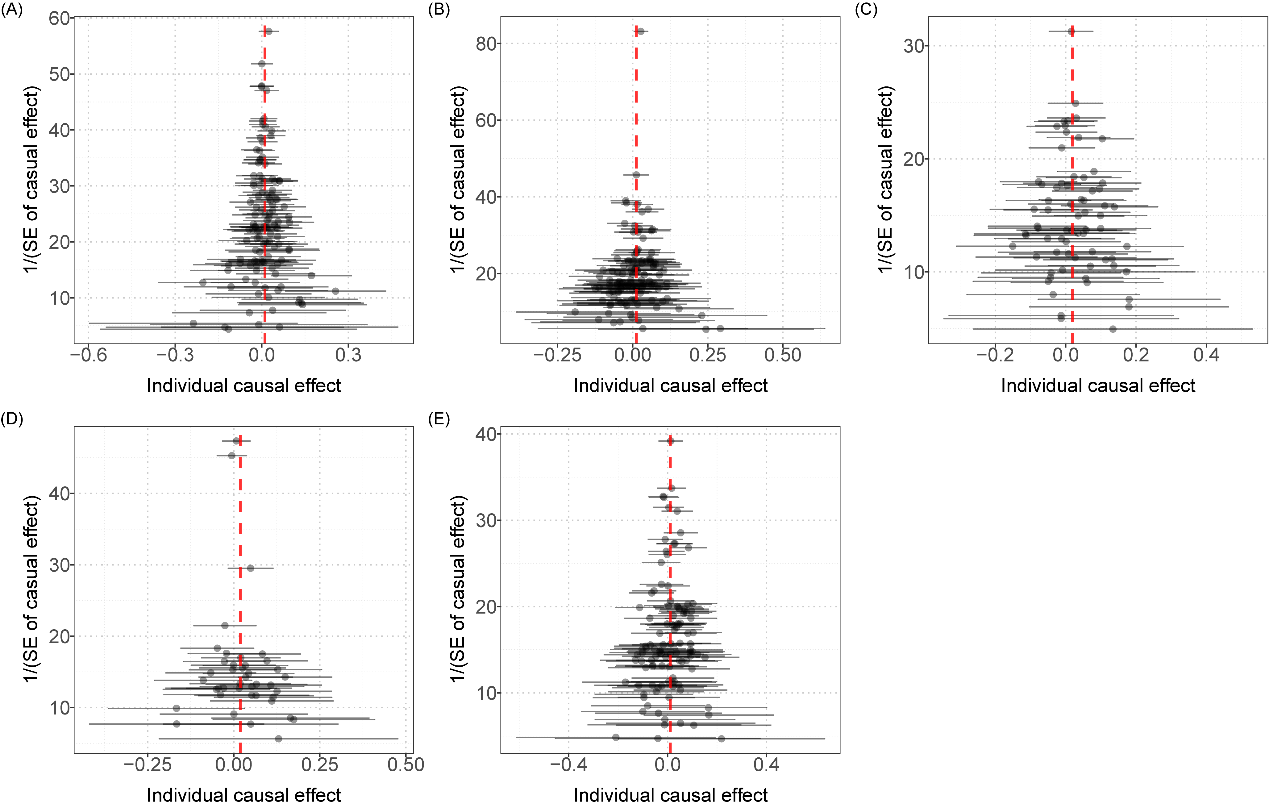


Figure S12. Funnel plot for the relationship between the SNP effect size of causal immune traits (MP) and the corresponding effect size estimates of BMDs. (A) SSC-A on monocyte on TB-BMD, (B) SSC-A on monocyte on FN-BMD, (C) SSC-A on CD14^+^ monocyte on LS-BMD, (D) SSC-A on myeloid DC on LS-BMD, (E) SSC-A on monocyte on LS-BMD. MP, morphological parameter; SSC, side scatter; TB, total body; FN, femoral neck; LS, lumbar spine, BMD, bone mineral density.


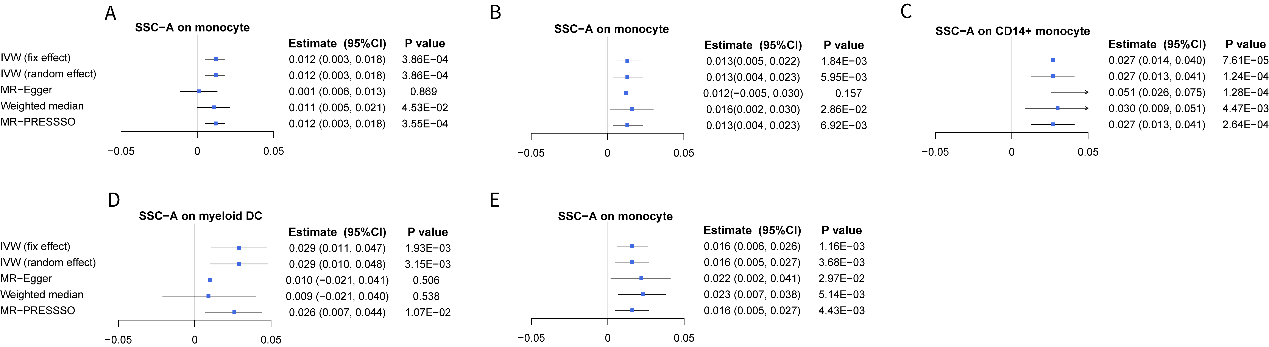


Figure S13. Forest plot of causal effects for different immune traits (MP) on BMDs. (A) SSC-A on monocyte on TB-BMD, (B) SSC-A on monocyte on FN-BMD, (C) SSC-A on CD14^+^ monocyte on LS-BMD, (D) SSC-A on myeloid DC on LS-BMD, (E) SSC-A on monocyte on LS-BMD. MP, morphological parameter; SSC, side scatter; TB, total body; FN, femoral neck; LS, lumbar spine, BMD, bone mineral density.


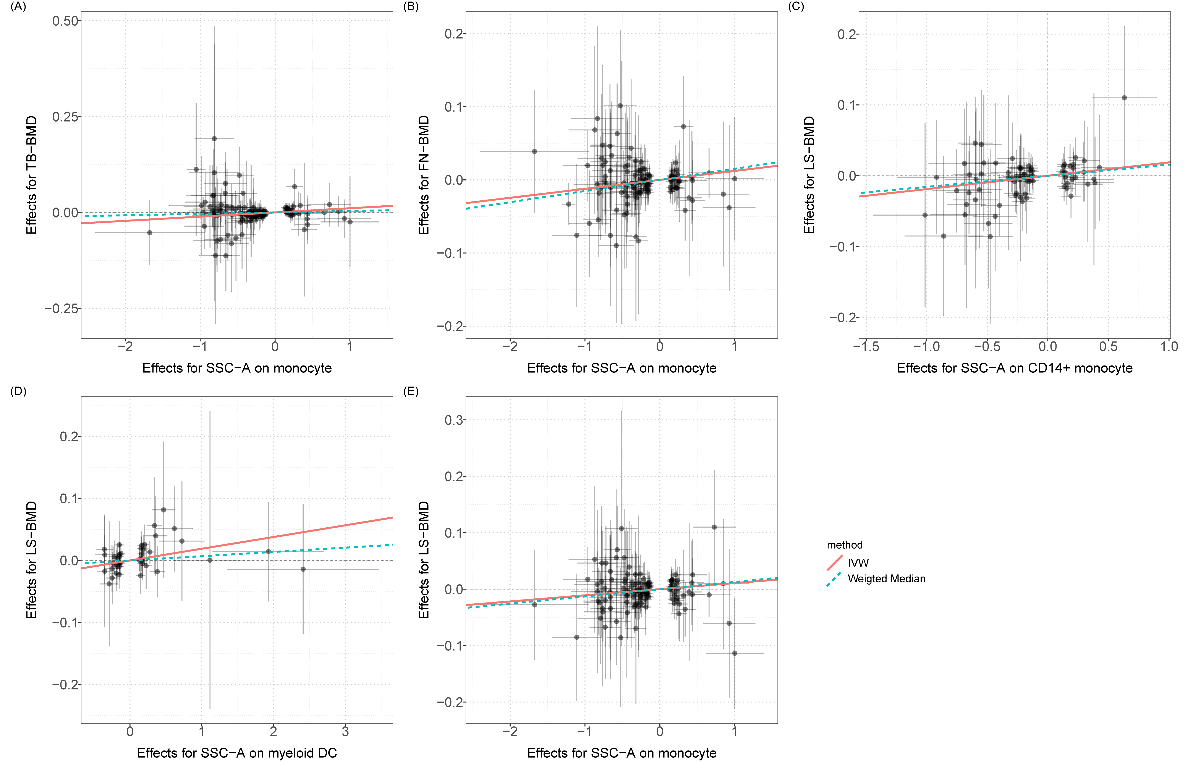


Figure S14. Scatter plot for the relationship between the SNP effect size of causal immune traits (x-axis) and the corresponding effect size estimates of BMDs (y-axis). (A) SSC-A on monocyte on TB-BMD, (B) SSC-A on monocyte on FN-BMD, (C) SSC-A on CD14^+^ monocyte on LS-BMD, (D) SSC-A on myeloid DC on LS-BMD, (E) SSC-A on monocyte on LS-BMD. MP, morphological parameter; SSC, side scatter; TB, total body; FN, femoral neck; LS, lumbar spine, BMD, bone mineral density.


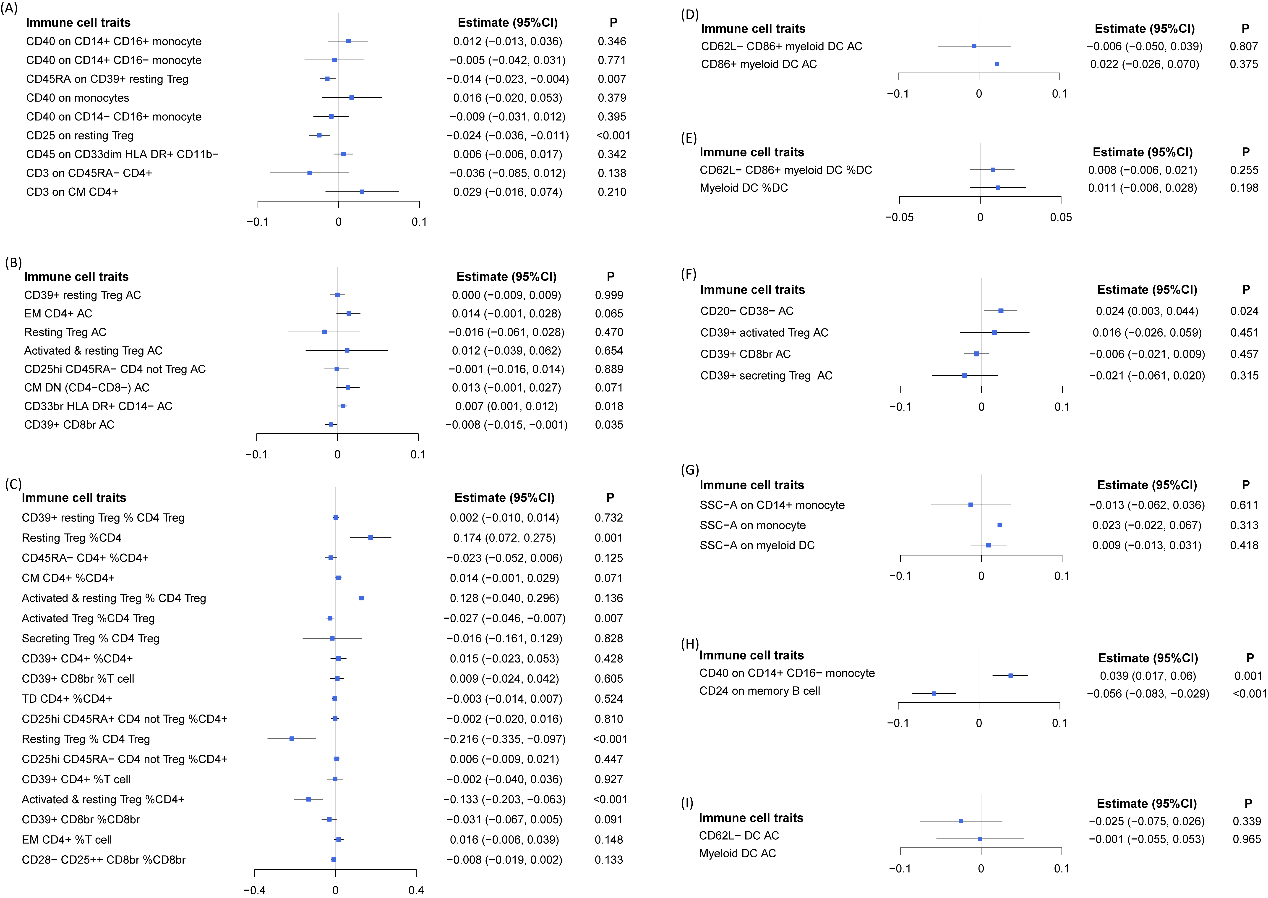


Figure S15. Effects of significant causal immune cell traits on different BMD traits estimated by using the multivariate MR regression. (A) TB-BMD (MFI), (B) TB-BMD (AC), (C) TB-BMD (RC), (D) FN-BMD (AC), (E) FN-BMD (RC), (F) LS-BMD (AC), (G) LS-BMD (MP), (H) FA-BMD (MFI), (I)FA-BMD (AC). MR: Mendelian randomization; TB, total body; FA, forearm; FN, femoral feck; LS, lumbar spine; BMD, bone mineral density; MFIs, median fluorescence intensities; AC, absolute count; RC, relative count; MP, morphological parameter.
