# Supplementary material for: The immune factors have complex causal regulation effects on bone mineral density
Source: Front Immunol. 2022 Oct 20;13:959417. doi: 10.3389/fimmu.2022.959417 (PMC9630477; doi:10.3389/fimmu.2022.959417)
Supplement: Supplementary file 1 [file DataSheet_1.zip › Data Sheet 1/959417-Supplementary-Material/Supplementary-Results.docx]

Supplementary Results

Robustness of results in the effects of immune traits (MFI) on BMDs

Additionally, the intercept of MR-Egger analysis for the association between several MFI traits (e.g., CD40 on CD14^+^ CD16^+^ monocyte and CD40 on monocytes) with BMD differed significantly from zero (*P* < 0.05), indicating some violations of the MR assumptions. After removing possible outliers detected by cook’s distance test, the causal effects of CD40 on CD14^+^ CD16^+^ monocyte (β = 0.023, *P*= 2.27E-09) and CD40 on monocytes (β = 0.019, *P* = 1.25E-06) were still significant and intercept of MR-Egger ruled out the possibility of horizontal pleiotropy (*P* = 0.122 for CD40 on CD14^+^ CD16^+^ monocytes and *P* = 0.739 for CD40 on monocytes) (**Table S7**), suggesting that an increased expression of CD40 on monocytes, which are precursors of osteoclast (multinucleated bone-resorbing cells) and DCs (mononuclear cells that initiate immune responses), may confer reduced risk for bone loss, although the mechanisms for this protection remain unclear. However, the causal effects of CD3 on central memory (CM) CD4^+^ (*P*= 0.645) and CD3 on CD45RA^-^ CD4^+^ (*P*= 0.184) were potentially biased by directional pleiotropy and became insignificant after removing outliers (**Table S7**). Although we cannot completely rule out any pleiotropy, the results of our MR analyses supported the particularly complex pattern displayed by the observed associations, where CD3 on both two subsets as well as the two subpopulations themselves presented distinct consequences on osteoporosis risk (**Table S3**).

Robustness of results in the effects of immune traits (RC) on BM

The MR-PRESSO global test (*P*= 1.130E-02) and intercept of MR-Egger analysis (*P*= 3.434E-02) suggested evidence of pleiotropy between TD CD4^+^ %CD4^+^ and TB-BMD. After removing several outliers by using cook’s distance method, the associations of this trait remained significant (β = -0.020, *P*= 1.09E-03) and the intercept of MR-Egger became unsignificant (*P* = 0.334) (**Table S7**). However, we also discovered the influences of CD3 expressed on CM CD4^+^ and CD45RA^-^ CD4^+^ traits were inconsistent with the effects of two traits in RC level (i.e., CM CD4^+^ %CD4^+^ and CD45RA^-^ CD4^+^ %CD4^+^) on BMD, and there was some horizontal pleiotropy for these two CD3 expressed traits in MFI types, indicating that CD3, a surface marker of a host of immune cells, may be complex when used as a therapeutic target.

Robustness of results in the effects of immune traits (MP) on BMDs

Additionally, we detected horizontal pleiotropy for SSC-A on CD14^+^ monocyte in LS-BMD (intercept = -0.007, *P* = 2.72E-02), the causal effects of several main MR methods still suggested a positive relationship between SSC-A on CD14^+^ monocyte and LS-BMD (intercept = -0.004, *P* = 0.239) after removing the outliers (**Table S7**). However, the intercept of MR-Egger analysis suggested the existence of uncontrolled pleiotropy that may bias the analyses of association between SSC-A on monocyte and TB-BMD (**Table S7**), even if we took measures to identify and exclude outliers. This pleiotropy has a very limited potential to influence the results as we performed several robust MR approaches.
