# Supplementary material for: The immune factors have complex causal regulation effects on bone mineral density
Source: Front Immunol. 2022 Oct 20;13:959417. doi: 10.3389/fimmu.2022.959417 (PMC9630477; doi:10.3389/fimmu.2022.959417)
Supplement: Supplementary file 1 [file DataSheet_1.zip › Data Sheet 1/959417-Supplementary-Material/Tables S3-S6-Captions.docx]

# Supplementary tables

Table S1. The instrumental variables for significant immune traits used in MR analyses.

Table S2. MR results of all immune traits identified in MR analysis.

Table S3. Estimated effect size of immune traits (MFI) on BMDs with different MR methods.

Table S4. Estimated effect size of immune traits (AC) on BMDs with different MR methods.

Table S5. Estimated effect size of immune traits (RC) on BMDs with different MR methods.

Table S6. Estimated effect size of immune traits (SSC) on BMDs with different MR methods.

Table S7. MR estimates from main methods after removing possible outliers.

Table S8. The inverse causal associations between immune traits and osteoporosis phenotypes.

Table S9. Mediation analysis of immune traits on osteoporosis with CD40 on monocytes.

**Table S3.** Estimated effect size of immune traits (MFI) on BMDs with different MR methods

| Variable (MFI) | Outcomes | N | MR Method | BETA (95%CI) | *p* | MR-PRESSO global test *p* | Heterogeneity  test *p* |
| --- | --- | --- | --- | --- | --- | --- | --- |
| CD40 on CD14^+^ CD16^+^ monocyte | TB-BMD | 90 | IVW (fixed effect) | 0.016 (0.010, 0.023) | 1.31E-06 | 0.608 | 0.609 |
|  |  |  | IVW (random effect) | 0.016 (0.010, 0.023) | 1.31E-06 |  |  |
|  |  |  | MR-Egger | 0.003 (-0.008, 0.014) | 0.564 |  |  |
|  |  |  | MR-Egger intercept | 0.006 (0.002, 0.009) | 2.078E-03 |  |  |
|  |  |  | Weighted median | 0.016 (0.005, 0.027) | 4.23E-03 |  |  |
|  |  |  | MR-PRESSO raw | 0.016 (0.010, 0.023) | 3.35E-06 |  |  |
| CD40 on CD14^+^ CD16^-^ monocyte | TB-BMD | 69 | IVW (fixed effect) | 0.018 (0.010, 0.026) | 8.63E-06 | 0.732 | 0.715 |
|  |  |  | IVW (random effect) | 0.018 (0.010, 0.026) | 8.63E-06 |  |  |
|  |  |  | MR-Egger | 0.004 (-0.001, 0.025) | 0.072 |  |  |
|  |  |  | MR-Egger intercept | 0.002 (0.002, 0.007) | 0.265 |  |  |
|  |  |  | Weighted median | 0.018 (0.005, 0.027) | 9.08E-03 |  |  |
|  |  |  | MR-PRESSO raw | 0.018 (0.010, 0.026) | 1.32E-05 |  |  |
| CD45RA on CD39^+^ resting Treg | TB-BMD | 37 | IVW (fixed effect) | -0.022 (-0.033, -0.012) | 3.61E-05 | 0.558 | 0.514 |
|  |  |  | IVW (random effect) | -0.022 (-0.033, -0.012) | 3.61E-05 |  |  |
|  |  |  | MR-Egger | 0.005 (-0.034, -0.005) | 1.12E-02 |  |  |
|  |  |  | MR-Egger intercept | -0.002 (0.003, 0.004) | 0.551 |  |  |
|  |  |  | Weighted median | -0.022 (0.005, 0.027) | 2.04E-03 |  |  |
|  |  |  | MR-PRESSO raw | -0.022 (-0.033, -0.012) | 1.74E-04 |  |  |
| CD40 on monocytes | TB-BMD | 102 | IVW (fixed effect) | 0.010 (0.004, 0.015) | 3.52E-04 | 0.623 | 0.742 |
|  |  |  | IVW (random effect) | 0.010 (0.004, 0.015) | 3.52E-04 |  |  |
|  |  |  | MR-Egger | 0.003 (-0.005, 0.010) | 0.443 |  |  |
|  |  |  | MR-Egger intercept | 0.004 (0.001, 0.007) | 6.111E-03 |  |  |
|  |  |  | Weighted median | 0.010 (0.005, 0.027) | 0.661 |  |  |
|  |  |  | MR-PRESSO raw | 0.010 (0.004, 0.015) | 2.89E-04 |  |  |
| CD40 on CD14^-^ CD16^+^ monocyte | TB-BMD | 116 | IVW (fixed effect) | 0.010 (0.004, 0.016) | 4.86E-04 | 0.849 | 0.837 |
|  |  |  | IVW (random effect) | 0.010 (0.004, 0.016) | 4.86E-04 |  |  |
|  |  |  | MR-Egger | 0.003 (0.005, 0.021) | 1.70E-03 |  |  |
|  |  |  | MR-Egger intercept | -0.001 (0.001, 0.001) | 0.341 |  |  |
|  |  |  | Weighted median | 0.010 (0.005, 0.027) | 6.64E-03 |  |  |
|  |  |  | MR-PRESSO raw | 0.010 (0.004, 0.016) | 2.91E-04 |  |  |
| CD45 on CD33^dim^ HLA DR^+^ CD11b^-^ | TB-BMD | 16 | IVW (fixed effect) | 0.041 (0.018, 0.063) | 3.65E-04 | 0.336 | 0.332 |
|  |  |  | IVW (random effect) | 0.041 (0.018, 0.063) | 7.52E-04 |  |  |
|  |  |  | MR-Egger | 0.011 (-0.048, 0.082) | 0.581 |  |  |
|  |  |  | MR-Egger intercept | 0.006 (0.007, 0.020) | 0.408 |  |  |
|  |  |  | Weighted median | 0.041 (0.005, 0.027) | 2.70E-03 |  |  |
|  |  |  | MR-PRESSO raw | 0.041 (0.018, 0.063) | 4.21E-03 |  |  |
| CD25 on resting Treg | TB-BMD | 32 | IVW (fixed effect) | -0.027 (-0.041, -0.013) | 2.12E-04 | 0.113 | 0.195 |
|  |  |  | IVW (random effect) | -0.027 (-0.041, -0.013) | 7.61E-04 |  |  |
|  |  |  | MR-Egger | 0.007 (-0.049, -0.003) | 2.72E-02 |  |  |
|  |  |  | MR-Egger intercept | 0.000 (0.003, 0.005) | 0.913 |  |  |
|  |  |  | Weighted median | -0.027 (0.005, 0.027) | 2.57E-02 |  |  |
|  |  |  | MR-PRESSO raw | -0.027 (-0.041, -0.013) | 7.84E-04 |  |  |
| CD24 on memory B cell | FA-BMD | 47 | IVW (fixed effect) | -0.052 (-0.077, -0.027) | 5.40E-05 | 0.275 | 0.318 |
|  |  |  | IVW (random effect) | -0.052 (-0.078, -0.026) | 1.08E-04 |  |  |
|  |  |  | MR-Egger | -0.054 (-0.091, -0.016) | 5.80E-03 |  |  |
|  |  |  | MR-Egger intercept | 0.001 (-0.010, 0.012) | 0.882 |  |  |
|  |  |  | Weighted median | -0.044 (-0.084, -0.003) | 3.58E-02 |  |  |
|  |  |  | MR-PRESSO raw | -0.052 (-0.079, -0.026) | 3.64E-04 |  |  |
| CD40 on CD14^+^ CD16^-^ monocyte | FA-BMD | 66 | IVW (fixed effect) | 0.041 (0.021, 0.061) | 4.87E-05 | 0.072 | 0.058 |
|  |  |  | IVW (random effect) | 0.041 (0.019, 0.064) | 3.48E-04 |  |  |
|  |  |  | MR-Egger | 0.023 (-0.013, 0.060) | 0.208 |  |  |
|  |  |  | MR-Egger intercept | 0.008 (-0.005, 0.020) | 0.215 |  |  |
|  |  |  | Weighted median | 0.041 (0.009, 0.072) | 1.20E-02 |  |  |
|  |  |  | MR-PRESSO raw | 0.041 (0.019, 0.064) | 6.63E-04 |  |  |

**Note:** MR: Mendelian randomization; N represents the number of SNPs used in different analysis. MFIs, median fluorescence intensities; TB-BMD, Total body bone mineral density; FN-BMD, Femoral Neck bone mineral density; LS-BMD, Lumbar Spine bone mineral density; FA-BMD, Forearm bone mineral density; IVW, inverse variance weighted method; MR-PRESSO global test refers to detection of horizontal pleiotropy; Heterogeneity test refers to Cochran's Q test; CI: Confidence interval.

**Table S4.** Estimated effect size of immune traits (AC) on BMDs with different MR methods

| Variable (AC) | Outcomes | N | MR Method | BETA (95%CI) | *p* | MR-PRESSO global test *p* | Heterogeneity  test *p* |
| --- | --- | --- | --- | --- | --- | --- | --- |
| CD39^+^ resting Treg AC | TB-BMD | 175 | IVW (fixed effect) | -0.011 (-0.016, -0.006) | 5.96E-06 | 0.254 | 0.244 |
|  |  |  | IVW (random effect) | -0.011 (-0.016, -0.006) | 1.23E-05 |  |  |
|  |  |  | MR-Egger | -0.014 (-0.022, -0.006) | 6.88E-04 |  |  |
|  |  |  | MR-Egger intercept | 0.001 (-0.002, 0.004) | 0.371 |  |  |
|  |  |  | Weighted median | -0.012 (-0.020, -0.004) | 3.49E-03 |  |  |
|  |  |  | MR-PRESSO raw | -0.011 (-0.016, -0.006) | 2.11E-05 |  |  |
| EM CD4^+^ AC | TB-BMD | 33 | IVW (fixed effect) | 0.021 (0.012, 0.031) | 1.93E-05 | 0.395 | 0.512 |
|  |  |  | IVW (random effect) | 0.021 (0.012, 0.031) | 1.93E-05 |  |  |
|  |  |  | MR-Egger | 0.017 (0.004, 0.030) | 1.02E-02 |  |  |
|  |  |  | MR-Egger intercept | 0.003 (-0.003, 0.009) | 0.283 |  |  |
|  |  |  | Weighted median | 0.017 (0.002, 0.032) | 2.95E-02 |  |  |
|  |  |  | MR-PRESSO raw | 0.021 (0.012, 0.031) | 1.04E-04 |  |  |
| Resting Treg AC | TB-BMD | 58 | IVW (fixed effect) | -0.021 (-0.031, -0.012) | 2.13E-05 | 0.182 | 0.503 |
|  |  |  | IVW (random effect) | -0.021 (-0.031, -0.012) | 2.13E-05 |  |  |
|  |  |  | MR-Egger | -0.030 (-0.044, -0.015) | 1.01E-04 |  |  |
|  |  |  | MR-Egger intercept | 0.004 (-0.001, 0.009) | 0.113 |  |  |
|  |  |  | Weighted median | -0.028 (-0.043, -0.012) | 4.01E-04 |  |  |
|  |  |  | MR-PRESSO raw | -0.019 (-0.029, -0.008) | 7.18E-04 |  |  |
| CD33^br^ HLA DR^+^ CD14^-^ AC | TB-BMD | 51 | IVW (fixed effect) | 0.008 (0.003, 0.014) | 2.00E-03 | 0.879 | 0.882 |
|  |  |  | IVW (random effect) | 0.008 (0.003, 0.014) | 2.00E-03 |  |  |
|  |  |  | MR-Egger | 0.006 (0.000, 0.012) | 4.97E-02 |  |  |
|  |  |  | MR-Egger intercept | 0.002 (-0.002, 0.006) | 0.235 |  |  |
|  |  |  | Weighted median | 0.005 (-0.003, 0.013) | 0.212 |  |  |
|  |  |  | MR-PRESSO raw | 0.008 (0.004, 0.013) | 9.22E-04 |  |  |
| CD25^hi^ CD45RA^-^ CD4 not Treg AC | TB-BMD | 40 | IVW (fixed effect) | 0.013 (0.005, 0.022) | 1.98E-03 | 0.191 | 0.193 |
|  |  |  | IVW (random effect) | 0.013 (0.004, 0.023) | 4.58E-03 |  |  |
|  |  |  | MR-Egger | 0.016 (0.003, 0.029) | 1.40E-02 |  |  |
|  |  |  | MR-Egger intercept | -0.003 (-0.010, 0.005) | 0.493 |  |  |
|  |  |  | Weighted median | 0.019 (0.005, 0.033) | 6.84E-03 |  |  |
|  |  |  | MR-PRESSO raw | 0.013 (0.004, 0.023) | 7.23E-03 |  |  |
| CD39^+^ CD8^br^ AC | TB-BMD | 245 | IVW (fixed effect) | -0.007 (-0.011, -0.002) | 3.46E-03 | 0.045 | 0.042 |
|  |  |  | IVW (random effect) | -0.007 (-0.011, -0.002) | 6.68E-03 |  |  |
|  |  |  | MR-Egger | -0.004 (-0.013, 0.004) | 0.310 |  |  |
|  |  |  | MR-Egger intercept | -0.001 (-0.004, 0.002) | 0.508 |  |  |
|  |  |  | Weighted median | -0.008 (-0.016, 0.001) | 0.071 |  |  |
|  |  |  | MR-PRESSO raw | -0.007 (-0.011, -0.002) | 7.16E-03 |  |  |
| Activated & resting Treg AC | TB-BMD | 27 | IVW (fixed effect) | -0.026 (-0.042, -0.010) | 1.72E-03 | 0.007 | 0.050 |
|  |  |  | IVW (random effect) | -0.026 (-0.046, -0.006) | 1.04E-02 |  |  |
|  |  |  | MR-Egger | -0.018 (-0.050, 0.014) | 0.263 |  |  |
|  |  |  | MR-Egger intercept | -0.003 (-0.011, 0.006) | 0.505 |  |  |
|  |  |  | Weighted median | -0.035 (-0.061, -0.009) | 7.56E-03 |  |  |
|  |  |  | MR-PRESSO raw | -0.020 (-0.042, 0.001) | 0.069 |  |  |
| CM DN (CD4^-^CD8^-^) AC | TB-BMD | 9 | IVW (fixed effect) | 0.027 (0.011, 0.043) | 8.62E-04 | 0.158 | 0.083 |
|  |  |  | IVW (random effect) | 0.027 (0.006, 0.048) | 1.16E-02 |  |  |
|  |  |  | MR-Egger | 0.028 (-0.005, 0.060) | 0.085 |  |  |
|  |  |  | MR-Egger intercept | -0.001 (-0.020, 0.019) | 0.934 |  |  |
|  |  |  | Weighted median | 0.021 (0.000, 0.043) | 0.055 |  |  |
|  |  |  | MR-PRESSO raw | 0.027 (0.006, 0.048) | 3.56E-02 |  |  |
| CD62L^-^ CD86^+^ myeloid DC AC | FN-BMD | 98 | IVW (fixed effect) | 0.015 (0.006, 0.024) | 5.63E-04 | 0.812 | 0.801 |
|  |  |  | IVW (random effect) | 0.015 (0.006, 0.024) | 5.63E-04 |  |  |
|  |  |  | MR-Egger | 0.009 (-0.006, 0.025) | 0.240 |  |  |
|  |  |  | MR-Egger intercept | 0.002 (-0.003, 0.007) | 0.396 |  |  |
|  |  |  | Weighted median | 0.016 (0.003, 0.030) | 1.33E-02 |  |  |
|  |  |  | MR-PRESSO raw | 0.015 (0.007, 0.023) | 3.80E-04 |  |  |
| CD86^+^ myeloid DC AC | FN-BMD | 78 | IVW (fixed effect) | 0.013 (0.004, 0.023) | 6.64E-03 | 0.934 | 0.925 |
|  |  |  | IVW (random effect) | 0.013 (0.004, 0.023) | 6.64E-03 |  |  |
|  |  |  | MR-Egger | 0.016 (-0.001, 0.033) | 0.067 |  |  |
|  |  |  | MR-Egger intercept | -0.001 (-0.006, 0.004) | 0.727 |  |  |
|  |  |  | Weighted median | 0.015 (0.000, 0.029) | 4.28E-02 |  |  |
|  |  |  | MR-PRESSO raw | 0.013 (0.005, 0.022) | 2.90E-03 |  |  |
| CD20^-^ CD38^-^ AC | LS-BMD | 21 | IVW (fixed effect) | 0.040 (0.016, 0.065) | 1.36E-03 | 0.777 | 0.673 |
|  |  |  | IVW (random effect) | 0.040 (0.016, 0.065) | 1.36E-03 |  |  |
|  |  |  | MR-Egger | 0.038 (0.005, 0.072) | 2.82E-02 |  |  |
|  |  |  | MR-Egger intercept | 0.001 (-0.008, 0.010) | 0.855 |  |  |
|  |  |  | Weighted median | 0.038 (0.002, 0.074) | 3.99E-02 |  |  |
|  |  |  | MR-PRESSO raw | 0.040 (0.019, 0.061) | 1.50E-03 |  |  |
| CD39^+^ activated Treg AC | LS-BMD | 191 | IVW (fixed effect) | -0.014 (-0.022, -0.005) | 1.30E-03 | 0.038 | 0.038 |
|  |  |  | IVW (random effect) | -0.014 (-0.023, -0.005) | 3.19E-03 |  |  |
|  |  |  | MR-Egger | -0.003 (-0.020, 0.015) | 0.760 |  |  |
|  |  |  | MR-Egger intercept | -0.003 (-0.008, 0.001) | 0.157 |  |  |
|  |  |  | Weighted median | -0.012 (-0.026, 0.003) | 0.109 |  |  |
|  |  |  | MR-PRESSO raw | -0.014 (-0.023, -0.005) | 3.59E-03 |  |  |
| CD39^+^ secreting Treg AC | LS-BMD | 219 | IVW (fixed effect) | -0.010 (-0.017, -0.003) | 4.11E-03 | 0.130 | 0.140 |
|  |  |  | IVW (random effect) | -0.010 (-0.017, -0.003) | 6.30E-03 |  |  |
|  |  |  | MR-Egger | -0.011 (-0.024, 0.002) | 0.087 |  |  |
|  |  |  | MR-Egger intercept | 0.000 (-0.004, 0.004) | 0.869 |  |  |
|  |  |  | Weighted median | -0.011 (-0.023, 0.002) | 0.090 |  |  |
|  |  |  | MR-PRESSO raw | -0.010 (-0.017, -0.003) | 6.82E-03 |  |  |
| CD39^+^ CD8^br^ AC | LS-BMD | 234 | IVW (fixed effect) | -0.010 (-0.016, -0.003) | 5.23E-03 | 0.200 | 0.200 |
|  |  |  | IVW (random effect) | -0.010 (-0.017, -0.003) | 7.12E-03 |  |  |
|  |  |  | MR-Egger | -0.009 (-0.021, 0.004) | 0.185 |  |  |
|  |  |  | MR-Egger intercept | 0.000 (-0.004, 0.004) | 0.851 |  |  |
|  |  |  | Weighted median | -0.007 (-0.019, 0.006) | 0.309 |  |  |
|  |  |  | MR-PRESSO raw | -0.010 (-0.017, -0.003) | 7.64E-03 |  |  |
| Myeloid DC AC | FA-BMD | 66 | IVW (fixed effect) | -0.027 (-0.046, -0.009) | 4.25E-03 | 0.438 | 0.418 |
|  |  |  | IVW (random effect) | -0.027 (-0.046, -0.008) | 4.77E-03 |  |  |
|  |  |  | MR-Egger | -0.014 (-0.041, 0.013) | 0.308 |  |  |
|  |  |  | MR-Egger intercept | -0.006 (-0.015, 0.002) | 0.158 |  |  |
|  |  |  | Weighted median | -0.011 (-0.042, 0.019) | 0.472 |  |  |
|  |  |  | MR-PRESSO raw | -0.027 (-0.046, -0.008) | 6.32E-03 |  |  |
| CD62L^-^ DC AC | FA-BMD | 68 | IVW (fixed effect) | -0.023 (-0.039, -0.007) | 5.98E-03 | 0.398 | 0.388 |
|  |  |  | IVW (random effect) | -0.023 (-0.040, -0.006) | 7.02E-03 |  |  |
|  |  |  | MR-Egger | -0.024 (-0.047, -0.001) | 3.94E-02 |  |  |
|  |  |  | MR-Egger intercept | 0.001 (-0.008, 0.009) | 0.892 |  |  |
|  |  |  | Weighted median | -0.016 (-0.045, 0.012) | 0.249 |  |  |
|  |  |  | MR-PRESSO raw | -0.023 (-0.040, -0.006) | 8.86E-03 |  |  |

**Note**: MR: Mendelian randomization; N represents the number of SNPs used in different analysis. AC, absolute count; TB-BMD, Total body bone mineral density; FN-BMD, Femoral Neck bone mineral density; LS-BMD, Lumbar Spine bone mineral density; FA-BMD, Forearm bone mineral density; IVW, inverse variance weighted method; MR-PRESSO global test refers to detection of horizontal pleiotropy; Heterogeneity test refers to Cochran's Q test; CI: Confidence interval.

**Table S5.** Estimated effect size of immune traits (RC) on BMDs with different MR methods

| Variable (RC) | Outcomes | N | MR Method | BETA (95%CI) | *p* | MR-PRESSO global test *p* | Heterogeneity  test *p* |
| --- | --- | --- | --- | --- | --- | --- | --- |
| Resting Treg %CD4 | TB-BMD | 86 | IVW (fixed effect) | -0.013 (-0.020, -0.007) | 6.38E-05 | 0.328 | 0.466 |
|  |  |  | IVW (random effect) | -0.013 (-0.020, -0.007) | 6.66E-05 |  |  |
|  |  |  | MR-Egger | -0.017 (-0.025, -0.009) | 6.41E-05 |  |  |
|  |  |  | MR-Egger intercept | 0.003 (-0.001, 0.006) | 0.106 |  |  |
|  |  |  | Weighted median | -0.016 (-0.027, -0.006) | 2.20E-03 |  |  |
|  |  |  | MR-PRESSO raw | -0.014 (-0.021, -0.007) | 8.083E-05 |  |  |
| CD39^+^ resting Treg % CD4 Treg | TB-BMD | 192 | IVW (fixed effect) | -0.007 (-0.011, -0.004) | 3.17E-05 | 0.184 | 0.186 |
|  |  |  | IVW (random effect) | -0.007 (-0.011, -0.004) | 6.77E-05 |  |  |
|  |  |  | MR-Egger | -0.008 (-0.012, -0.003) | 1.06E-03 |  |  |
|  |  |  | MR-Egger intercept | 0.000 (-0.002, 0.002) | 0.838 |  |  |
|  |  |  | Weighted median | -0.004 (-0.011, 0.002) | 0.164 |  |  |
|  |  |  | MR-PRESSO raw | -0.007 (-0.011, -0.004) | 9.63E-05 |  |  |
| CM CD4^+^ %CD4^+^ | TB-BMD | 49 | IVW (fixed effect) | 0.012 (0.006, 0.018) | 1.65E-04 | 0.773 | 0.753 |
|  |  |  | IVW (random effect) | 0.012 (0.006, 0.018) | 1.65E-04 |  |  |
|  |  |  | MR-Egger | 0.009 (0.002, 0.016) | 1.55E-02 |  |  |
|  |  |  | MR-Egger intercept | 0.003 (0.000, 0.007) | 0.071 |  |  |
|  |  |  | Weighted median | 0.010 (0.000, 0.020) | 0.055 |  |  |
|  |  |  | MR-PRESSO raw | 0.012 (0.006, 0.018) | 1.70E-04 |  |  |
| CD45RA^-^ CD4^+^ %CD4^+^ | TB-BMD | 55 | IVW (fixed effect) | 0.016 (0.004, 0.025) | 2.97E-04 | 0.096 | 0.199 |
|  |  |  | IVW (random effect) | 0.016 (0.005, 0.026) | 7.74E-04 |  |  |
|  |  |  | MR-Egger | 0.018 (0.006, 0.030) | 6.83E-03 |  |  |
|  |  |  | MR-Egger intercept | -0.001 (0.002, 0.004) | 0.717 |  |  |
|  |  |  | Weighted median | 0.020 (0.007, 0.034) | 7.11E-03 |  |  |
|  |  |  | MR-PRESSO raw | 0.015 (0.005, 0.024) | 4.50E-03 |  |  |
| Activated & resting Treg % CD4 Treg | TB-BMD | 87 | IVW (fixed effect) | -0.011 (0.003, -0.005) | 4.41E-04 | 0.130 | 0.114 |
|  |  |  | IVW (random effect) | -0.011 (0.003, -0.004) | 1.26E-03 |  |  |
|  |  |  | MR-Egger | -0.015 (0.004, -0.006) | 7.49E-04 |  |  |
|  |  |  | MR-Egger intercept | 0.002 (0.002, 0.006) | 0.142 |  |  |
|  |  |  | Weighted median | -0.018 (0.005, -0.009) | 1.76E-04 |  |  |
|  |  |  | MR-PRESSO raw | -0.011 (0.003, -0.005) | 1.46E-03 |  |  |
| CD39^+^ CD4^+^ %CD4^+^ | TB-BMD | 290 | IVW (fixed effect) | -0.007 (0.002, -0.003) | 9.89E-04 | 0.209 | 0.199 |
|  |  |  | IVW (random effect) | -0.007 (0.002, -0.003) | 1.45E-03 |  |  |
|  |  |  | MR-Egger | -0.006 (0.004, 0.002) | 0.143 |  |  |
|  |  |  | MR-Egger intercept | 0.000 (0.001, 0.002) | 0.700 |  |  |
|  |  |  | Weighted median | -0.007 (0.005, 0.002) | 0.104 |  |  |
|  |  |  | MR-PRESSO raw | -0.007 (-0.012, -0.003) | 1.61E-03 |  |  |
| CD39^+^ CD8^br^ %T cell | TB-BMD | 254 | IVW (fixed effect) | -0.007 (0.002, -0.003) | 1.59E-03 | 0.140 | 0.139 |
|  |  |  | IVW (random effect) | -0.007 (0.002, -0.003) | 2.57E-03 |  |  |
|  |  |  | MR-Egger | -0.008 (0.004, 0.000) | 0.060 |  |  |
|  |  |  | MR-Egger intercept | 0.000 (0.001, 0.003) | 0.786 |  |  |
|  |  |  | Weighted median | -0.008 (0.005, 0.002) | 0.107 |  |  |
|  |  |  | MR-PRESSO raw | -0.007 (0.002, -0.003) | 2.83E-03 |  |  |
| Secreting Treg % CD4 Treg | TB-BMD | 90 | IVW (fixed effect) | 0.010 (0.003, 0.017) | 9.46E-04 | 0.088 | 0.083 |
|  |  |  | IVW (random effect) | 0.010 (0.003, 0.017) | 2.69E-03 |  |  |
|  |  |  | MR-Egger | 0.014 (0.004, 0.023) | 1.57E-03 |  |  |
|  |  |  | MR-Egger intercept | -0.002 (0.002, 0.001) | 0.162 |  |  |
|  |  |  | Weighted median | 0.015 (0.005, 0.025) | 2.80E-03 |  |  |
|  |  |  | MR-PRESSO raw | 0.011 (0.003, 0.017) | 2.91E-03 |  |  |
| CD25^hi^ CD45RA^+^ CD4 not Treg %CD4^+^ | TB-BMD | 74 | IVW (fixed effect) | -0.009 (0.003, -0.004) | 3.85E-04 | 0.013 | 0.009 |
|  |  |  | IVW (random effect) | -0.009 (0.003, -0.003) | 3.05E-03 |  |  |
|  |  |  | MR-Egger | -0.011 (0.004, -0.004) | 2.90E-03 |  |  |
|  |  |  | MR-Egger intercept | 0.002 (0.002, 0.007) | 0.293 |  |  |
|  |  |  | Weighted median | -0.011 (0.004, -0.004) | 3.90E-03 |  |  |
|  |  |  | MR-PRESSO raw | -0.009 (0.003, -0.003) | 4.11E-03 |  |  |
| Activated Treg %CD4 Treg | TB-BMD | 42 | IVW (fixed effect) | 0.017 (0.005, 0.028) | 1.01E-03 | 0.138 | 0.125 |
|  |  |  | IVW (random effect) | 0.017 (0.006, 0.029) | 3.37E-03 |  |  |
|  |  |  | MR-Egger | 0.023 (0.008, 0.038) | 6.14E-03 |  |  |
|  |  |  | MR-Egger intercept | -0.003 (0.003, 0.003) | 0.311 |  |  |
|  |  |  | Weighted median | 0.023 (0.008, 0.039) | 4.43E-03 |  |  |
|  |  |  | MR-PRESSO raw | 0.017 (0.006, 0.029) | 5.48E-03 |  |  |
| Resting Treg % CD4 Treg | TB-BMD | 120 | IVW (fixed effect) | -0.009 (0.003, -0.004) | 8.64E-04 | 0.010 | 0.008 |
|  |  |  | IVW (random effect) | -0.009 (0.003, -0.003) | 3.96E-03 |  |  |
|  |  |  | MR-Egger | -0.012 (0.004, -0.005) | 1.94E-03 |  |  |
|  |  |  | MR-Egger intercept | 0.003 (0.002, 0.006) | 0.164 |  |  |
|  |  |  | Weighted median | -0.015 (0.004, -0.006) | 1.02E-03 |  |  |
|  |  |  | MR-PRESSO raw | -0.009 (-0.015, -0.003) | 4.09E-03 |  |  |
| CD39^+^ CD4^+^ %T cell | TB-BMD | 274 | IVW (fixed effect) | -0.006 (0.002, -0.002) | 4.63E-03 | 0.387 | 0.379 |
|  |  |  | IVW (random effect) | -0.006 (0.002, -0.002) | 5.14E-03 |  |  |
|  |  |  | MR-Egger | -0.006 (0.003, 0.000) | 0.059 |  |  |
|  |  |  | MR-Egger intercept | 0.000 (0.001, 0.002) | 0.903 |  |  |
|  |  |  | Weighted median | -0.007 (0.004, 0.000) | 0.060 |  |  |
|  |  |  | MR-PRESSO raw | -0.006 (-0.010, -0.002) | 5.50E-03 |  |  |
| EM CD4^+^ %T cell | TB-BMD | 38 | IVW (fixed effect) | 0.015 (0.005, 0.025) | 5.71E-03 | 0.062 | 0.462 |
|  |  |  | IVW (random effect) | 0.015 (0.005, 0.025) | 5.81E-03 |  |  |
|  |  |  | MR-Egger | 0.016 (0.007, 0.030) | 2.74E-02 |  |  |
|  |  |  | MR-Egger intercept | -0.001 (0.003, 0.005) | 0.776 |  |  |
|  |  |  | Weighted median | 0.014 (0.008, 0.030) | 0.078 |  |  |
|  |  |  | MR-PRESSO raw | 0.008 (0.006, 0.019) | 0.171 |  |  |
| CD39^+^ CD8^br^ %CD8^br^ | TB-BMD | 241 | IVW (fixed effect) | -0.006 (0.002, -0.002) | 5.83E-03 | 0.132 | 0.126 |
|  |  |  | IVW (random effect) | -0.006 (0.002, -0.002) | 8.72E-03 |  |  |
|  |  |  | MR-Egger | -0.008 (0.004, 0.001) | 0.073 |  |  |
|  |  |  | MR-Egger intercept | 0.000 (0.001, 0.003) | 0.719 |  |  |
|  |  |  | Weighted median | -0.008 (0.005, 0.002) | 0.105 |  |  |
|  |  |  | MR-PRESSO raw | -0.006 (-0.011, -0.002) | 9.27E-03 |  |  |
| TD CD4^+^ %CD4^+^ | TB-BMD | 26 | IVW (fixed effect) | -0.024 (0.007, -0.009) | 1.45E-03 | 0.011 | 0.051 |
|  |  |  | IVW (random effect) | -0.024 (0.009, -0.006) | 9.39E-03 |  |  |
|  |  |  | MR-Egger | -0.045 (0.013, -0.019) | 1.68E-03 |  |  |
|  |  |  | MR-Egger intercept | 0.007 (0.003, 0.013) | 3.434E-02 |  |  |
|  |  |  | Weighted median | -0.038 (0.011, -0.016) | 7.09E-04 |  |  |
|  |  |  | MR-PRESSO raw | -0.018 (0.007, -0.005) | 9.46E-03 |  |  |
| CD25^hi^ CD45RA^-^ CD4 not Treg %CD4^+^ | TB-BMD | 46 | IVW (fixed effect) | 0.013 (0.005, 0.023) | 4.57E-03 | 0.142 | 0.134 |
|  |  |  | IVW (random effect) | 0.013 (0.005, 0.024) | 1.07E-02 |  |  |
|  |  |  | MR-Egger | 0.019 (0.007, 0.033) | 8.99E-03 |  |  |
|  |  |  | MR-Egger intercept | -0.003 (0.003, 0.002) | 0.232 |  |  |
|  |  |  | Weighted median | 0.021 (0.008, 0.037) | 1.10E-02 |  |  |
|  |  |  | MR-PRESSO raw | 0.013 (0.003, 0.024) | 1.34E-02 |  |  |
| Activated & resting Treg %CD4^+^ | TB-BMD | 62 | IVW (fixed effect) | -0.012 (0.004, -0.004) | 5.68E-03 | 0.118 | 0.162 |
|  |  |  | IVW (random effect) | -0.012 (0.005, -0.003) | 1.08E-02 |  |  |
|  |  |  | MR-Egger | -0.010 (0.006, 0.003) | 0.114 |  |  |
|  |  |  | MR-Egger intercept | -0.001 (0.002, 0.003) | 0.691 |  |  |
|  |  |  | Weighted median | -0.020 (0.007, -0.006) | 6.01E-03 |  |  |
|  |  |  | MR-PRESSO raw | -0.011 (-0.020, -0.001) | 2.86E-02 |  |  |
| CD28^-^ CD25^++^ CD8^br^ %CD8^br^ | TB-BMD | 35 | IVW (fixed effect) | -0.021 (0.008, -0.005) | 1.19E-02 | 0.532 | 0.699 |
|  |  |  | IVW (random effect) | -0.021 (0.008, -0.005) | 1.19E-02 |  |  |
|  |  |  | MR-Egger | -0.014 (0.012, 0.010) | 0.245 |  |  |
|  |  |  | MR-Egger intercept | -0.002 (0.003, 0.003) | 0.473 |  |  |
|  |  |  | Weighted median | -0.025 (0.013, 0.000) | 4.63E-02 |  |  |
|  |  |  | MR-PRESSO raw | -0.020 (0.008, -0.005) | 1.41E-02 |  |  |
| CD62L^-^ CD86^+^ myeloid DC %DC | FN-BMD | 101 | IVW (fixed effect) | 0.014 (0.006, 0.023) | 8.49E-04 | 0.953 | 0.952 |
|  |  |  | IVW (random effect) | 0.014 (0.006, 0.023) | 8.49E-04 |  |  |
|  |  |  | MR-Egger | 0.014 (-0.001, 0.028) | 0.064 |  |  |
|  |  |  | MR-Egger intercept | 0.000 (-0.004, 0.005) | 0.900 |  |  |
|  |  |  | Weighted median | 0.019 (0.006, 0.032) | 3.96E-03 |  |  |
|  |  |  | MR-PRESSO raw | 0.014 (0.007, 0.022) | 2.61E-04 |  |  |
| Myeloid DC %DC | FN-BMD | 57 | IVW (fixed effect) | 0.017 (0.006, 0.028) | 2.10E-03 | 1.000 | 1.000 |
|  |  |  | IVW (random effect) | 0.017 (0.006, 0.028) | 2.10E-03 |  |  |
|  |  |  | MR-Egger | 0.020 (0.004, 0.036) | 1.24E-02 |  |  |
|  |  |  | MR-Egger intercept | -0.001 (-0.006, 0.003) | 0.605 |  |  |
|  |  |  | Weighted median | 0.022 (0.005, 0.039) | 1.13E-02 |  |  |
|  |  |  | MR-PRESSO raw | 0.017 (0.010, 0.025) | 4.37E-05 |  |  |
| Myeloid DC %DC | LS-BMD | 57 | IVW (fixed effect) | 0.022 (0.009, 0.035) | 8.23E-04 | 0.236 | 0.229 |
|  |  |  | IVW (random effect) | 0.022 (0.008, 0.036) | 1.68E-03 |  |  |
|  |  |  | MR-Egger | 0.023 (0.002, 0.044) | 2.88E-02 |  |  |
|  |  |  | MR-Egger intercept | 0.000 (-0.006, 0.005) | 0.896 |  |  |
|  |  |  | Weighted median | 0.020 (-0.001, 0.041) | 0.062 |  |  |
|  |  |  | MR-PRESSO raw | 0.022 (0.008, 0.036) | 2.69E-03 |  |  |
| CD14^+^ CD16^+^ monocyte %monocyte | FA-BMD | 77 | IVW (fixed effect) | 0.037 (0.014, 0.060) | 1.80E-03 | 0.802 | 0.797 |
|  |  |  | IVW (random effect) | 0.037 (0.014, 0.060) | 1.80E-03 |  |  |
|  |  |  | MR-Egger | 0.023 (-0.025, 0.071) | 0.343 |  |  |
|  |  |  | MR-Egger intercept | 0.004 (-0.008, 0.017) | 0.505 |  |  |
|  |  |  | Weighted median | 0.023 (-0.014, 0.061) | 0.226 |  |  |
|  |  |  | MR-PRESSO raw | 0.037 (0.015, 0.059) | 1.22E-03 |  |  |

**Note**: MR: Mendelian randomization; N represents the number of SNPs used in different analysis. RC, relative count; TB-BMD, Total body bone mineral density; FN-BMD, Femoral Neck bone mineral density; LS-BMD, Lumbar Spine bone mineral density; FA-BMD, Forearm bone mineral density; IVW, inverse variance weighted method; MR-PRESSO global test refers to detection of horizontal pleiotropy; Heterogeneity test refers to Cochran's Q test; CI: Confidence interval.

**Table S6.** Estimated effect size of immune traits (SSC) on BMDs with different MR methods

| Variable (SSC) | Outcomes | N | MR Method | BETA (95%CI) | *p* | MR-PRESSO global test *p* | Heterogeneity  test *p* |
| --- | --- | --- | --- | --- | --- | --- | --- |
| SSC-A on monocyte | TB-BMD | 122 | IVW (fixed effect) | 0.012 (0.003, 0.018) | 3.86E-04 | 0.703 | 0.690 |
|  |  |  | IVW (random effect) | 0.012 (0.003, 0.018) | 3.86E-04 |  |  |
|  |  |  | MR-Egger | 0.001 (0.006, 0.013) | 0.869 |  |  |
|  |  |  | MR-Egger intercept | 0.004 (0.002, 0.007) | 2.74E-02 |  |  |
|  |  |  | Weighted median | 0.011 (0.005, 0.021) | 4.53E-02 |  |  |
|  |  |  | MR-PRESSO raw | 0.012 (0.003, 0.018) | 3.55E-04 |  |  |
| SSC-A on monocyte | FN-BMD | 115 | IVW (fixed effect) | 0.013 (0.005, 0.022) | 1.84E-03 | 0.024 | 0.023 |
|  |  |  | IVW (random effect) | 0.013 (0.004, 0.023) | 5.95E-03 |  |  |
|  |  |  | MR-Egger | 0.012 (-0.005, 0.030) | 0.157 |  |  |
|  |  |  | MR-Egger intercept | 0.000 (-0.005, 0.005) | 0.894 |  |  |
|  |  |  | Weighted median | 0.016 (0.002, 0.030) | 2.86E-02 |  |  |
|  |  |  | MR-PRESSO raw | 0.013 (0.004, 0.023) | 6.92E-03 |  |  |
| SSC-A on CD14^+^ monocyte | LS-BMD | 73 | IVW (fixed effect) | 0.027 (0.014, 0.040) | 7.61E-05 | 0.327 | 0.336 |
|  |  |  | IVW (random effect) | 0.027 (0.013, 0.041) | 1.24E-04 |  |  |
|  |  |  | MR-Egger | 0.051 (0.026, 0.075) | 1.28E-04 |  |  |
|  |  |  | MR-Egger intercept | -0.007 (-0.013, -0.001) | 2.72E-02 |  |  |
|  |  |  | Weighted median | 0.030 (0.009, 0.051) | 4.47E-03 |  |  |
|  |  |  | MR-PRESSO raw | 0.027 (0.013, 0.041) | 2.64E-04 |  |  |
| SSC-A on myeloid DC | LS-BMD | 34 | IVW (fixed effect) | 0.029 (0.011, 0.047) | 1.93E-03 | 0.171 | 0.313 |
|  |  |  | IVW (random effect) | 0.029 (0.010, 0.048) | 3.15E-03 |  |  |
|  |  |  | MR-Egger | 0.010 (-0.021, 0.041) | 0.506 |  |  |
|  |  |  | MR-Egger intercept | 0.006 (-0.002, 0.014) | 0.125 |  |  |
|  |  |  | Weighted median | 0.009 (-0.021, 0.040) | 0.538 |  |  |
|  |  |  | MR-PRESSO raw | 0.026 (0.007, 0.044) | 1.07E-02 |  |  |
| SSC-A on monocyte | LS-BMD | 115 | IVW (fixed effect) | 0.016 (0.006, 0.026) | 1.16E-03 | 0.033 | 0.036 |
|  |  |  | IVW (random effect) | 0.016 (0.005, 0.027) | 3.68E-03 |  |  |
|  |  |  | MR-Egger | 0.022 (0.002, 0.041) | 2.97E-02 |  |  |
|  |  |  | MR-Egger intercept | -0.002 (-0.008, 0.004) | 0.489 |  |  |
|  |  |  | Weighted median | 0.023 (0.007, 0.038) | 5.14E-03 |  |  |
|  |  |  | MR-PRESSO raw | 0.016 (0.005, 0.027) | 4.43E-03 |  |  |

**Note:** MR: Mendelian randomization; N represents the number of SNPs used in different analysis. SSC, side scatter; TB-BMD, Total body bone mineral density; FN-BMD, Femoral Neck bone mineral density; LS-BMD, Lumbar Spine bone mineral density; FA-BMD, Forearm bone mineral density; IVW, inverse variance weighted method; MR-PRESSO global test refers to detection of horizontal pleiotropy; Heterogeneity test refers to Cochran's Q test; CI: Confidence interval.
